# Genome-wide association study of susceptibility to acute respiratory distress syndrome

**DOI:** 10.1016/j.ebiom.2025.105951

**Published:** 2025-09-30

**Authors:** Beatriz Guillen-Guio, Eva Suarez-Pajes, Eva Tosco-Herrera, Tamara Hernandez-Beeftink, Jose Miguel Lorenzo-Salazar, Diana Chang, Rafaela González-Montelongo, Luis A. Rubio-Rodríguez, Olivia C. Leavy, Richard J. Allen, Almudena Corrales, Raquel Cruz, Miguel Bardají-Carrillo, Angel Carracedo, Eduardo Tamayo, V. Eric Kerchberger, Lorraine B. Ware, Brian L. Yaspan, Markus Scholz, André Scherag, Jesús Villar, Louise V. Wain, Carlos Flores

**Affiliations:** a Department of Population Health Sciences, University of Leicester, Leicester, United Kingdom; b NIHR Leicester Biomedical Research Centre, Leicester, United Kingdom; c Centro de Investigación Biomédica en Red de Enfermedades Respiratorias (CIBERES), Instituto de Salud Carlos III, Madrid, Spain; d Research Unit, Hospital Universitario Nuestra Señora de Candelaria, Instituto de Investigación Sanitaria de Canarias, Santa Cruz de Tenerife, Spain; e Genomics Division, Instituto Tecnológico y de Energías Renovables, Santa Cruz de Tenerife, Spain; f Department of Human Genetics, Genentech, CA, USA; g Centro Singular de Investigación en Medicina Molecular y Enfermedades Crónicas (CIMUS), Universidade de Santiago de Compostela, Santiago de Compostela, Spain; h Centre for Biomedical Network Research on Rare Diseases (CIBERER), Instituto de Salud Carlos III, Madrid, Spain; i Fundación Pública Galega de Medicina Xenómica, Sistema Galego de Saúde (SERGAS) Santiago de Compostela, Santiago de Compostela, Spain; j Department of Anesthesiology and Critical Care, Hospital Clínico Universitario de Valladolid, Valladolid, Spain; k Instituto de Investigación Sanitaria de Santiago (IDIS), Santiago de Compostela, Spain; l Biomedicine Group in Critical Care (BioCritic), Spain; m Centro de Investigación Biomédica en Red de Enfermedades Infecciosas (CIBERINFEC), Instituto de Salud Carlos III, Madrid, Spain; n Department of Medicine, Vanderbilt University School of Medicine, Nashville, USA; o Department of Biomedical Informatics, Vanderbilt University Medical Center, Nashville, USA; p Institute for Medical Informatics, Statistics and Epidemiology, University of Leipzig, Leipzig, Germany; q Institute of Medical Statistics, Computer and Data Sciences, Jena University Hospital, Jena, Germany; r Research Unit, Hospital Universitario Dr. Negrín, Fundación Canaria Instituto de Investigación Sanitaria de Canarias, Las Palmas de Gran Canaria, Spain; s Li Ka Shing Knowledge Institute, St. Michael's Hospital, Toronto, Canada; t Faculty of Health Sciences, Universidad del Atlántico Medio, Las Palmas, Spain; u Facultad de Ciencias de la Salud, Universidad Fernando Pessoa Canarias, Las Palmas de Gran Canaria, Spain

**Keywords:** ARDS, Cholesterol metabolism, GWAS, Sepsis, Statin

## Abstract

**Background:**

Acute respiratory distress syndrome (ARDS) is a severe inflammatory process of the lung, often due to sepsis, and poses significant mortality burden in intensive care units. Here we conducted a genome-wide association study (GWAS) of ARDS to identify genetic risk loci that can help guide the development of new therapeutic options.

**Methods:**

We performed a case–control GWAS in 716 cases with ARDS, mainly associated with severe infections, and 4399 at-risk controls from three independent studies. Results were meta-analysed across the three studies, with significance set at *p* < 5 × 10^−8^. Suggestive associations were declared for variants exhibiting consistent direction of effects, likely to replicate and nominal significance (*p* < 0.05) in all three studies. Prioritised loci were subjected to Bayesian fine mapping, *in-silico* functional assessments, and gene-based rare variant collapsing analysis using whole-exome sequencing data. Two independent studies with 430 ARDS cases and 1398 at-risk controls served as validation samples.

**Findings:**

We identified a variant near *HMGCR* that showed genome-wide significant association with ARDS and had been previously linked to cholesterol metabolism. This locus was associated with *ANKDD1B* expression in artery. The rare exonic variant analysis showed associations between *HMGCR* and ARDS at nominal level (*p* < 0.05). While no nominal significance was achieved in the two additional validation cohorts, this variant exhibited a consistent direction of effects across all 5 studies.

**Interpretation:**

A common variant near *HMGCR* was associated with ARDS risk, suggesting a link between cholesterol metabolism and ARDS risk. Validation in independent studies is needed.

**Funding:**

10.13039/100010269Wellcome Trust, National Institute for Health Research Leicester Biomedical Research Centre, 10.13039/100000050National Heart, Lung, and Blood Institute, ATS Research Program, Gobierno de Canarias, 10.13039/501100023554Fundación Canaria Instituto de Investigación Sanitaria de Canarias, 10.13039/100017487Instituto Tecnológico y de Energías Renovables, Cabildo Insular de Tenerife, 10.13039/501100004587Instituto de Salud Carlos III, 10.13039/501100011033Agencia Estatal de Investigación, German Ministry of Education and Research, 10.13039/501100004404Thuringian Ministry of Education, Science and Culture, the Thuringian Foundation for Technology, Innovation, and Research, German Sepsis Society.

**Research in context**

**Evidence before this study**

Acute respiratory distress syndrome (ARDS) is a critical lung condition characterised by a severe inflammatory response and respiratory failure. Patients require immediate treatment in an intensive care unit, where there is a lack of specific therapeutic options. Genetic studies can improve our understanding of the syndrome, providing advances for patient management and detecting therapeutic targets.

**Added value of this study**

The study examined the genetic association in 716 ARDS patients and 4399 at-risk controls and prioritised nine genetic regions of interest. Combined with complementary approaches and rare exonic variation analyses, the results supported *HMGCR* as a gene of interest for ARDS.

**Implications of all the available evidence**

Our results support the link between cholesterol metabolism and ARDS risk and underline the value of applying genomic approaches for the understanding of ARDS.

## Introduction

Acute respiratory distress syndrome (ARDS) is a severe lung condition with an overall hospital mortality of about 40%.^1^ It develops due to injury to the capillary alveolar membrane, which can be triggered by direct or indirect causes, including pulmonary and non-pulmonary sepsis, severe pneumonia, major trauma, and blood transfusion, among others.^2^ ARDS is characterised by a rapid onset of lung inflammatory damage, resulting in hypoxaemia and acute respiratory failure. Furthermore, ARDS survivors frequently manifest long-term complications, including pulmonary fibrosis development. The diagnosis of ARDS still relies on clinical and imaging criteria.^3^

ARDS is a medical emergency that requires immediate management in intensive care units (ICUs). The treatment of ARDS primarily involves supportive care such as mechanical ventilation to improve oxygenation, prone positioning, and medications to address underlying causes and manage symptoms. There is a lack of specific lung-directed pharmacological treatments with demonstrated benefit for ARDS patients in clinical trials.^4^ Thus, seeking effective treatments and specific prognostic methods is crucial for enhancing the survival outcomes of ARDS patients.

Leveraging genomic information could hold the key for guiding the search for future ARDS treatments, as it supports clinical trial efficiency by identifying drug targets with genetic support.^5^ The genetics of ARDS is complex and not yet fully understood. However, there is evidence supporting the central role of host genetic factors in the development and severity of ARDS.^6^ Genome-wide association study (GWAS) approaches have been used to identify genetic variants contributing to the risk of this syndrome typically by comparing genetic variation between cases and controls. We recently completed a GWAS of sepsis-associated ARDS in a two-stage study comprising 1935 ICU patients (633 ARDS cases and 1302 controls with sepsis).^7^ This analysis identified a genome-wide significant locus associated with ARDS susceptibility in a regulatory region of the promoter region of the *FLT1* gene, encoding the vascular endothelial growth factor receptor 1.

Here we aimed to detect additional genetic loci involved in ARDS to improve our understanding of the syndrome, by meta-analysing case–control genotype data from three independent studies, followed by the integration of exome sequencing data and findings from two additional studies.

## Methods

### Study design

**Fig. 1:**
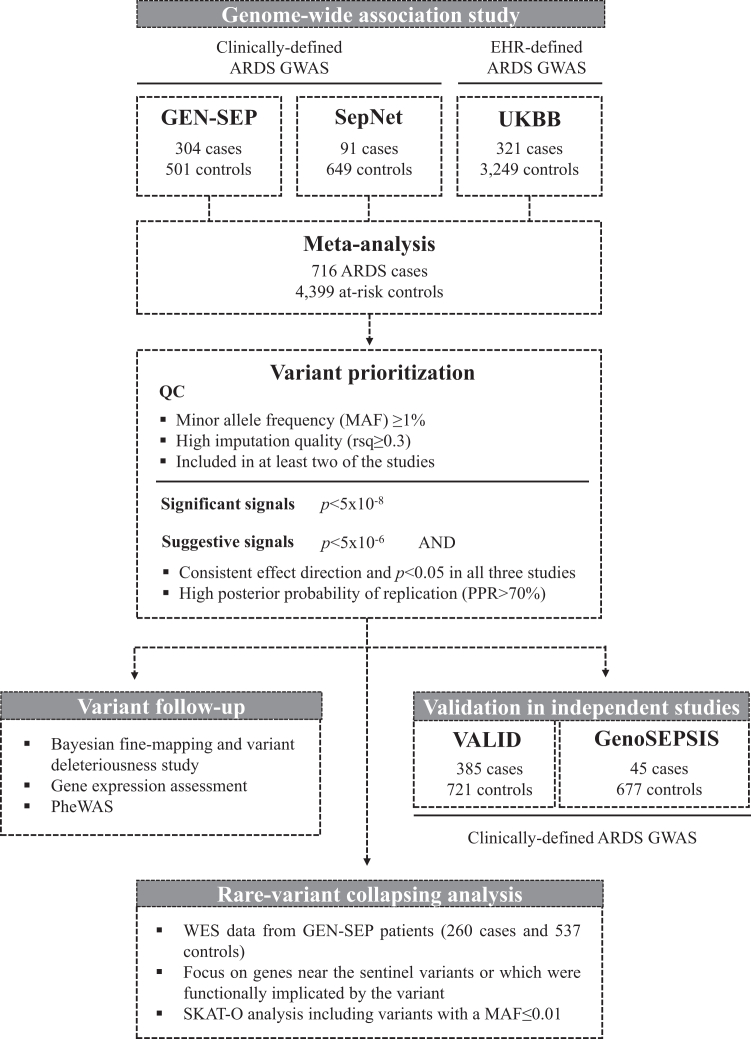
**Flowchart of the study design**. EHR: Electronic Health Records; PheWAS: Phenome-Wide Association Study; QC: Quality Controls; UKBB: UK Biobank; WES: Whole-exome sequencing.

We performed a GWAS meta-analysis on 5115 critically ill patients, 82.8% with severe infections, comprising 716 ARDS cases and 4399 at-risk controls from three independent studies (Fig. 1, Table S1): the GENetics of SEPsis-induced ARDS Network (GEN-SEP), the Critical Care Trials Group of the German Sepsis Competence Network (SepNet), and UK Biobank (UKBB). All individuals from the GEN-SEP and SepNet cohorts were considered in the analyses. Because these cohorts did not include COVID-19 patients, all non-COVID-19 ARDS cases from the UK Biobank were selected for the UKBB GWAS. For the UKBB control group, ten individuals per ARDS case were selected to ensure a statistical power greater than 74% (see power calculation in the Appendix). We included a total of 805 patients from the GEN-SEP cohort (304 ARDS cases and 501 at-risk controls), 740 patients from SepNet (91 ARDS cases and 649 at-risk controls), and 3570 patients from UKBB (321 ARDS cases and 3249 at-risk controls). Of these, 1330 patients were included in the prior GWAS (590 from GEN-SEP and 740 from SepNet).^7^ The GEN-SEP and SepNet studies comprised cases of clinically defined sepsis-associated ARDS (Berlin definition)^2^ and controls with sepsis who did not develop ARDS. The UKBB study was based on electronic health record data, encompassing 75.4% of patients with ARDS associated with ICD-10 codes indicative of severe infections (septicaemia and/or pneumonia) (n = 242) and 24.6% with ARDS associated with other causes (n = 79). The code J80 was used to define ARDS. ICD-10 codes used to determine the ARDS development causes are provided in Table S2. UKBB controls were critically ill patients who did not develop ARDS (75.4% with ICD-10 codes indicative of septicaemia and/or pneumonia). All individuals from the GEN-SEP and SepNet cohorts were of European ancestry, while more than 92% of individuals in UKBB were of European ancestry (Table S3). ICD-10 codes were not available for the GEN-SEP and SepNet cohorts. Further details of the three studies and the design are provided in the Appendix.

### GWAS meta-analysis and statistical analyses

A GWAS of ARDS was performed independently in each of the three studies. Participants whose genetic sex differed from their self-reported sex were excluded from the analyses. Logistic regression models assumed an additive inheritance. Genotyping, quality control and association testing procedures are described in the Appendix.

The results of the three GWAS were meta-analysed across the GEN-SEP, SepNet, and UKBB studies using a fixed-effect inverse-variance weighted meta-analysis in METAL (2011 version)^8^ for all autosomal variants present in at least two studies. The assessment of chromosome X was limited to the GEN-SEP and UKBB studies. To identify independent associated variants, we performed conditional regression analyses using the GCTA COnditional and JOint association analysis using GWAS summary statistics tool (GCTA-COJO)^9^ around 1 Mb of the sentinel variant in the prioritised loci, considering the underlying linkage disequilibrium (LD) structure in the study sample. Variant prioritisation was performed based on the replicability of the association signals using the Meta-Analysis Model-Based Assessment of Replicability (MAMBA).^10^ MAMBA calculates the posterior probability of replicability (PPR) that a given variant has a non-zero replicable effect across the three studies, indicating the likelihood of a specific genetic variant to replicate.

Genome-wide significant associations were declared for variants satisfying a *p* < 5 × 10^−8^ in the first-stage meta-analysis. We also declared suggestive associations for variants satisfying all the following criteria: *p* < 5 × 10^−6^, consistent effect direction and *p* < 0.05 in all studies with available data, and a MAMBA PPR ≥ 70% in the meta-analysis (considered consistent and likely to replicate) (Fig. 1). Both significant and suggestive loci were prioritised for subsequent analyses. For these loci, METAL was used to assess the effect size differences across studies with the Cochran's Q for heterogeneity.^8^ If deemed significant (*p* < 0.05), an additive random-effects model was also applied using the DerSimonian-Laird estimator. In addition, we also examined our previously reported ARDS protective variant at *FLT1* (rs9508032, chr13:28995940) revealed in the GEN-SEP and SepNet studies^7^ and a variant at *BORCS5* (rs7967111, chr12:12601953) reported in a multi-ancestry GWAS.^11^

We performed Bayesian fine-mapping on the association results around the sentinel variants on each prioritised associated locus to identify the credible set of variants that most likely harbours the causal one with 95% confidence. Posterior probabilities (PP) were calculated from approximate Bayes factors for all variants within a region of 1 Mb and a r^2^ > 0.1 with the sentinel variant.^12^ Variants were considered part of the credible set until their sum of probabilities was >0.95 (see the Appendix for details).

### *In-silico* functional assessment

We aimed to identify the most biologically relevant variants included in the credible sets. First, we functionally annotated the variants using Ensembl Variant Effect Predictor (VEP) v.105 to obtain the scaled Combined Annotation Dependent Depletion (CADD) score v.1.6 of each variant. Second, variants with the highest phenotypic impact, based on their CADD score, within each identified credible set were further investigated through a phenome-wide association study (PheWAS) using publicly available data from Open Targets^13^ to evaluate their pleiotropic effect and identify previous associations with other phenotypes. We set a significance threshold at *p* < 1 × 10^−5^ after Bonferroni correction for the number of tested phenotypes. Finally, we used the Genotype-Tissue Expression (GTEx) Release v.8 data^14^ to evaluate the existence of expression quantitative trait loci (eQTL) in tissues of interest for the disease (i.e. lung, cultured fibroblasts, whole blood, thyroid, tibial and aorta artery, Epstein–Barr virus-transformed lymphocytes and oesophagus mucosa). The significance threshold was set at *p* < 6.25 × 10^−3^ after Bonferroni correction for the eight tissues. We performed colocalisation analysis using the R package *coloc* under a single causal variant assumption^15^ to study whether the same causal variant was driving both genetic association with ARDS and gene expression. Additional information is described in the Appendix.

### Rare-variant collapsing analysis on prioritised loci

We accessed whole-exome sequencing (WES) data from 260 patients with sepsis-associated ARDS and 537 at-risk controls with sepsis from the GEN-SEP cohort (see Appendix for more details). WES analyses focused on genes near the significant or suggestively associated loci and those resulting from colocalisation analysis. To assess the association of gene-based rare exonic variation, we used the optimal sequence kernel association test (SKAT-O) implemented in EPACTS v.3.6.2.^16^^,^^17^ Testing was performed separately for two categories of variants: (i) all variants with MAF ≤ 0.01, and (ii) all MAF ≤ 0.01 variants classified as likely having high phenotypic impact (see Appendix). Analyses were controlled for sex, age, and APACHE II, and significance was established at *p* < 1.79 × 10^−3^ considering tests for two categories of variants in 14 genes.

### Validation in independent studies

Following the initial meta-analysis, investigators from two independent studies of patients with sepsis were contacted, and their data were used to validate the prioritised associations (Fig. 1). The first study included 1106 patients of European ancestry (385 sepsis-associated ARDS cases and 721 sepsis controls without ARDS) from the prospective study Validating Acute Lung Injury biomarkers for Diagnosis (VALID).^18^ Cases and controls were required to have sepsis and organ dysfunction. Diagnosis of ARDS in VALID was done by two-physician review using the Berlin definition. The second study consisted of 45 ARDS cases (Berlin definition) and 677 at-risk controls (primarily with septic shock) from the Genomic Study of Sepsis (GenoSEPSIS).^19^ Further details of the two studies, including genotyping, imputation and association testing, are described in the Appendix. Results from the GWAS meta-analysis and validation studies were meta-analysed with METAL following the same procedure as that of the first-stage meta-analysis.

### Ethics

The GEN-SEP study was approved by the Ethics Committee from the Hospital Universitario de Canarias (CHUNSC_2018-16 and CHUNSC_2021-40). Genetic analyses of SepNet (with separate patient consent for genetic analyses) were integrated in the randomised clinical trials VISEP (recruitment in 2003–2005, registration number: NCT00135473) and MAXSEP (recruitment in 2007–2010, registration number: NCT00534287), approved by the Ethics Committee at each participating institution. UK Biobank has approval from the North West Multi-centre Research Ethics Committee (MREC). VALID and GenoSEPSIS studies were approved by their corresponding Research Ethics Committees (Vanderbilt Institutional Review Board (IRB) #051065 for VALID and PI 20-2070 for GenoSEPSIS). All these participating studies were performed according to The Code of Ethics of the World Medical Association (Declaration of Helsinki), and written informed consent was obtained from all subjects or their representatives. For VALID, a waiver of consent was approved when the patient was unable to provide consent and no surrogate decision maker could be identified.

### Role of funders

The funders of the study had no role in study design, data collection, data analysis, data interpretation, or writing of the manuscript.

## Results

**Fig. 2:**
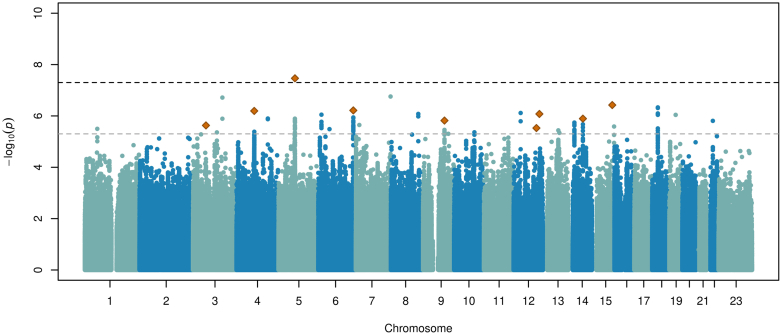
**Manhattan plot of the GWAS meta-analysis results**. The y-axis shows the transformed *p*-values (−log10 *p*-value), and the x-axis represents the chromosome positions (GRCh37/hg19). Genome-wide significance (*p*-value = 5.0 × 10^−8^) and suggested significance (*p*-value = 5.0 × 10^−6^) thresholds are indicated by the upper and lower horizontal dashed lines, respectively. The X chromosome is represented as 23 and contains the meta-analysis results of GEN-SEP and UKBB. The orange diamond represents the prioritised variants (inflation λ = 0.99).

**Fig. 3:**
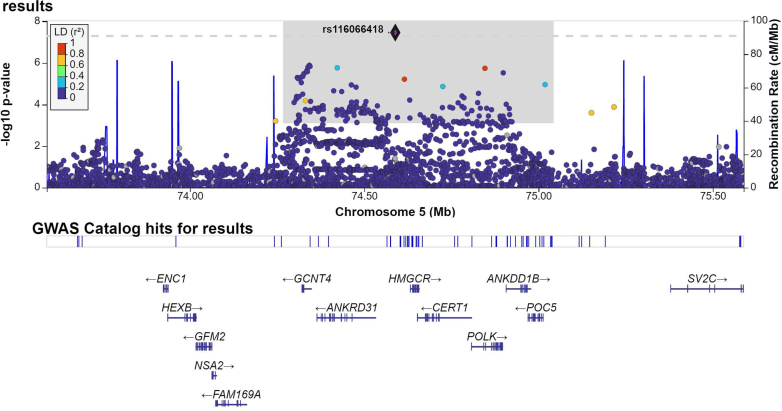
**Regional plot of the association results for the genome-wide significant variant at 5q13.3**. The y-axis shows the transformed *p*-values (−log10 [*p*-value]), and the x-axis represents the chromosome positions (GRCh37/hg19). Genome-wide significance threshold (*p*-value = 5.0 × 10^−8^) is indicated by the horizontal dashed line. Linkage disequilibrium (LD) values (r^2^) are based on the European population data from The 1000 Genomes Project and are represented according to the LD colour scheme of the top left legend. The shaded grey area denotes the variants contained within the credible set. The plot was generated with LocusZoom (http://locuszoom.org/).

**Fig. 4:**
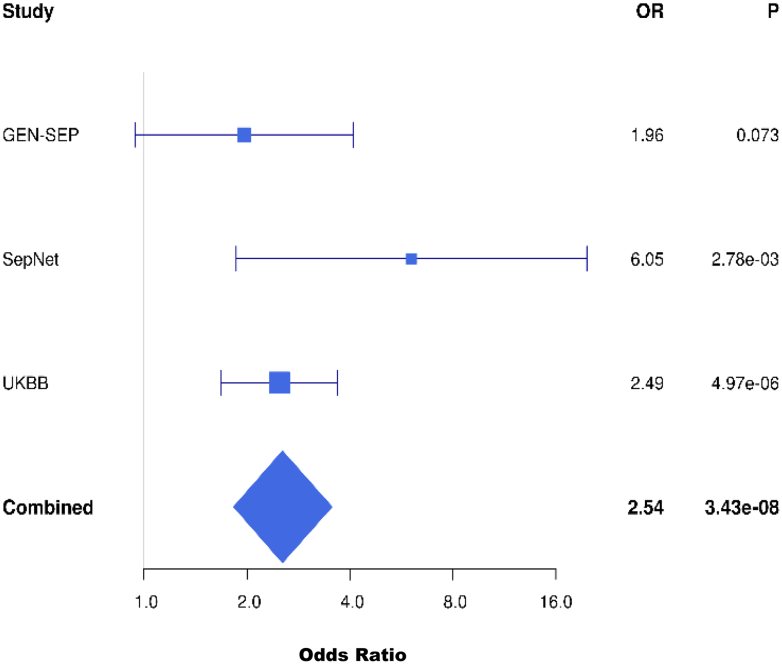
**Forest plot of the association results for the genome-wide significant variant at 5q13.3 showing odds ratios with 95% confidence intervals for each study**.

A total of 716 ARDS cases and 4399 at-risk controls, and 8,469,305 SNPs with a MAF > 0.01, high imputation quality and present in at least two of the three studies were included in the first-stage meta-analysis. There was no evidence of inflation (λ = 0.99) (Figure S1). A variant at chromosome 5q13.3 near *HMGCR* was genome-wide significantly associated with ARDS with a high probability of replication (rs116066418, 5q13.3, MAF = 0.023, odds ratio (OR) = 2.54, 95% confidence interval (CI) = 1.83–3.54, *p* = 3.43 × 10^−8^, PPR = 0.99) (Table 1, Fig. 2, Fig. 3, Fig. 4, Figure S2). Results were robust after excluding non-European individuals (OR (95% CI) = 2.57 (1.84–3.58), *p* = 2.50 × 10^−8^, PPR = 0.99). A further eight loci showed suggestive significance (*p* < 5 × 10^−6^) and high probability of replication (MAMBA PPR ≥ 70%), as well as significance at the nominal level (*p* < 0.05) and consistent effect direction in all three studies contributing to the meta-analysis (Figures S2 and S3). The *p*-value for heterogeneity statistics exceeded 0.05 for all of the prioritised variants except one (rs11111647, Cochran's Q-test *p* = 0.048), suggesting that there are no differences in effect sizes across studies. For rs11111647, the *p*-value decreased to 3.17 × 10^−3^ when an additive random-effects model was applied using the DerSimonian-Laird estimator, although the effect size remained similar (OR (95% CI) = 1.50 (1.15–1.96)). The nine independent loci (one genome-wide significant and eight suggestively significant) were prioritised for further analyses. Association results remained consistent after excluding patients without sepsis or pneumonia, who accounted for 17.20% of the study sample (Table S4). The association of the variant at *FLT1* identified in our previous GWAS of sepsis-associated ARDS (rs9508032, chr13:28995940), which used data from GEN-SEP and SepNet studies,^7^ was not associated with ARDS in the UKBB study (*p* = 0.809), including after restricting the analysis to patients with septicaemia and/or pneumonia (*p* = 0.955) (Figure S4, Table S5). The intronic variant at *BORCS5* (rs7967111, chr12:12601953) revealed in the ARDS GWAS performed by Du and colleagues^11^ was not associated with ARDS in our study either (*p* = 0.622) (Figure S4).

**Table 1:** Association analysis results for the nine signals prioritised after meta-analysis of the three ARDS studies.

ID	CHR. band	POS (b37)	Nearest gene/s	NEA/EA	EA_freq	OR (95% CI)	*p*-value	PPR	Direction^a^
rs749263563	3p14.2	3:58794870	*FAM3D*	TAGAC/T	0.023	2.66 (1.77, 4.00)	2.34 × 10^−6^	0.96	?++
rs183861060	4q21.21	4:80403211	*GK2*	A/G	0.014	2.95 (1.93, 4.52)	6.39 × 10^−7^	0.90	+?+
rs116066418	5q13.3	5:74588898	*ANKRD31, HMGCR*	T/A	0.023	2.54 (1.83, 3.54)	3.43 × 10^−8^	0.99	+++
rs59685452	6q25.3	6:159277046	*EZR*	G/C	0.030	2.12 (1.58, 2.86)	6.10 × 10^−7^	0.98	+++
rs12684841	9q22.32	9:97408971	*FBP1, AOPEP*	A/G	0.079	1.63 (1.34, 1.99)	1.51 × 10^−6^	0.90	+++
rs11111647	12q23.3	12:103945224	*STAB2*	G/A	0.212	1.40 (1.22, 1.62)	2.97 × 10^−6^	0.75	+++
rs118026254	12q24.22	12:117415150	*FBXW8*	G/C	0.033	2.18 (1.60, 2.96)	8.38 × 10^−7^	0.82	+?+
rs8022645	14q23.3	14:66327149	*FUT8*, *CCDC196*	C/T	0.234	1.40 (1.22, 1.61)	1.28 × 10^−6^	0.76	+++
rs4989808	15q26.1	15:92466080	*SLCO3A1*	A/G	0.020	2.66 (1.82, 3.87)	3.77 × 10^−7^	0.94	+?+

CHR: chromosome, CI: confidence interval, EA: effect allele, EA_freq: frequency of the effect allele, NEA: non-effect allele, OR: Odds ratio, POS: position, PPR: posterior probability of replication (obtained with MAMBA, Meta-Analysis Model-Based Assessment of Replicability).

a Order for effect Direction is: GEN-SEP—SepNet—UKBB (+ means beta >0 and ? means missing data).

Bayesian genetic fine-mapping around each of the nine loci was performed to identify the most likely causal variants driving the association. The 95% credible sets were provided for all the loci except the one at chromosome 15q26.1, due to the absence of LD with the sentinel variant in this region (Tables S6 and S7, Figure S5). For each locus, we selected the variant with the highest CADD score, revealing three variants with a high phenotypic impact prediction (CADD>15.00) within the credible sets of the loci at chromosomes 3p14.2 (rs80308704, CADD = 17.80), 5q13.3 (rs6893216, CADD = 16.66), and 9q22.32 (rs73523269, CADD = 15.78) (Tables S6 and S7).

*In-silico* functional evaluation revealed that the risk allele of the 5q13.3 missense variant rs6893216 was associated with increased *POC5* expression in cultured fibroblasts, thyroid and Epstein–Barr Virus-transformed lymphocytes, and with increased *ANKDD1B* expression in artery (Table S7). The ARDS GWAS signal colocalised with *ANKDD1B* eQTL signals in tibial artery (colocalisation probability [coloc] >70%). PheWAS results revealed that the risk allele of this variant has also been associated with increased LDL and total cholesterol levels (*p* = 7.8 × 10^−115^ and *p* = 4.7 × 10^−67^, respectively), increased statin medication (*p* = 1.8 × 10^−14^), and higher platelet count (*p* = 1.2 × 10^−8^), amongst others (Table S7). The risk allele of the variant at 9q22.32 was associated with reduced expression of *FBP1* in oesophagus, where the causal variant driving ARDS risk and *FBP1* expression was the same (coloc = 88.7%). The variant at 3p14.2 did not emerge as a significant eQTL for any genes and has not been previously associated with other traits at genome-wide level (Table S7).

The gene-based rare exonic variant analysis revealed a nominal association (*p* < 0.05) between *HMGCR* and ARDS when all variants with MAF < 0.01 were considered in the analysis (*p* = 0.038), and between *POC5* and ARDS when all variants with MAF < 0.01 and a likely high phenotypic impact were included (*p* = 0.011) (Table 2). These two genes are near or functionally implicated by the genome wide significant variant at 5q13.3.

**Table 2:** Results of the rare exonic gene–level associations at prioritised loci.

Locus	Gene^a^	Chr	Start-end	Test	Variants^b^	*p*-value
3p14.2	*FAM3D*	3	58,619,673–58,652,575	MAF < 0.01	7	0.165
				MAF < 0.01 | high-impact	5	0.068
4q21.21	*GK2*	4	80,327,508–80,329,372	MAF < 0.01	11	0.105
				MAF < 0.01 | high-impact	9	0.128
5q13.3	*ANKRD31*	5	74,364,100–74,532,703	MAF < 0.01	27	1.000
				MAF < 0.01 | high-impact	15	1.000
	*HMGCR*	5	74,632,154–74,657,929	MAF < 0.01	9	**0.038**
				MAF < 0.01 | high-impact	7	0.142
	*ANKDD1B*	5	74,907,284–74,967,671	MAF < 0.01	10	0.125
				MAF < 0.01 | high-impact	2	0.145
	*POC5*	5	74,969,949–75,013,313	MAF < 0.01	9	0.163
				MAF < 0.01 | high-impact	8	**0.011**
6q25.3	*EZR*	6	159,186,773–159,240,444	MAF < 0.01	7	0.714
				MAF < 0.01 | high-impact	6	0.329
9q22.32	*MFSD14B*	9	97,136,833–97,223,324	MAF < 0.01	8	0.688
				MAF < 0.01 | high-impact	6	0.579
	*FBP1*	9	97,365,415–97,402,531	MAF < 0.01	3	0.298
				MAF < 0.01 | high-impact	3	0.289
	*AOPEP*	9	97,488,983–97,849,441	MAF < 0.01	17	0.306
				MAF < 0.01 | high-impact	13	0.109
12q23.3	*STAB2*	12	103,981,051–104,160,505	MAF < 0.01	59	0.879
				MAF < 0.01 | high-impact	52	0.915
12q24.22	*FBXW8*	12	117,348,761–117,468,953	MAF < 0.01	17	0.778
				MAF < 0.01 | high-impact	14	0.727
14q23.3	*FUT8*	14	65,877,310–66,210,839	MAF < 0.01	4	0.383
				MAF < 0.01 | high-impact	4	0.384
15q26.1	*SLCO3A1*	15	92,396,925–92,715,665	MAF < 0.01	6	0.071
				MAF < 0.01 | high-impact	5	0.127

Chr: Chromosome; MAF: Minor Allele Frequency. Gene positions based on GRCh37/hg19, Ensembl.

Bold values indicate results that are statistically significant at the nominal level (*p* < 0.05).

a Genes near the sentinel variants or which were functionally implicated by the highest phenotypic impact SNP in each locus.

b Number of variants tested. Results were not available for *CCDC196*.

Finally, the leading independent variants from the nine prioritised loci were tested for association with ARDS in VALID and GenoSEPSIS cohorts. While none of the variants reached nominal significance (*p* < 0.05) in either independent validation cohorts, rs11111647 (12q23.3) became slightly more significant after the second-stage meta-analysis, and three variants had consistent direction of effects across the five studies, including the genome-wide significant variant at 5q13.3 and variants located at the 6q25.3 and 12q23.3 loci (Table 3).

**Table 3:** Association results in the validation cohorts.

ID (POS b37)	NEA/EA	First-stage meta-analysis (716 cases/4399 controls)		VALID (385 cases/721 controls)		GenoSEPSIS (45 cases/677 controls)		Second-stage meta-analysis (1146 cases/5797 controls)	
		OR (95% CI)	*p*-value	OR (95% CI)	*p*-value	OR (95% CI)	*p*-value	OR (95% CI)	*p*-value
**rs749263563** (3:58794870, 3p14.2)	TAGAC/T	2.66 (1.77, 4.00)	2.34 × 10^−6^	0.85 (0.48, 1.53)	0.593	NA	NA	1.83 (1.31, 2.56)	3.74 × 10^−4^
**rs183861060** (4:80403211, 4q21.21)	A/G	2.95 (1.93, 4.52)	6.39 × 10^−7^	1.08 (0.69, 1.69)	0.749	0.78 (0.18, 3.45)	0.740	1.77 (1.31, 2.40)	2.19 × 10^−4^
**rs116066418** (5:74588898, 5q13.3)	T/A	2.54 (1.83, 3.54)	3.43 × 10^−8^	1.28 (0.73, 2.25)	0.391	1.22 (0.26, 5.65)	0.802	2.09 (1.58, 2.77)	2.49 × 10^−7^
**rs59685452** (6:159277046, 6q25.3)	G/C	2.12 (1.58, 2.86)	6.10 × 10^−7^	1.01 (0.55, 1.85)	0.972	1.78 (0.66, 4.80)	0.255	1.84 (1.42, 2.38)	3.38 × 10^−6^
**rs12684841** (9:97408971, 9q22.32)	A/G	1.63 (1.34, 1.99)	1.51 × 10^−6^	0.97 (0.70, 1.34)	0.846	1.46 (0.71, 3.00)	0.300	1.42 (1.20, 1.67)	3.78 × 10^−5^
**rs11111647** (12:103945224, 12q23.3)	G/A	1.40 (1.22, 1.62)	2.97 × 10^−6^	1.10 (0.89, 1.36)	0.378	1.62 (1.00, 2.65)	0.051	1.32 (1.17, 1.48)	2.91 × 10^−6^
**rs118026254** (12:117415150, 12q24.22)	G/C	2.18 (1.60, 2.96)	8.38 × 10^−7^	1.14 (0.71, 1.83)	0.596	0.41 (0.09, 1.80)	0.239	1.72 (1.33, 2.22)	3.48 × 10^−5^
**rs8022645** (14:66327149, 14q23.3)	C/T	1.40 (1.22, 1.61)	1.28 × 10^−6^	0.97 (0.79, 1.20)	0.792	1.05 (0.63, 1.76)	0.844	1.25 (1.12, 1.40)	1.11 × 10^−4^
**rs4989808** (15:92466080, 15q26.1)	A/G	2.66 (1.82, 3.87)	3.77 × 10^−7^	1.01 (0.59, 1.73)	0.967	0.66 (0.08, 5.28)	0.698	1.89 (1.39, 2.56)	4.35 × 10^−5^

CI: confidence interval, EA: effect allele, NEA: non-effect allele, NA: results not available in the cohort, as it does not include indels, OR: Odds ratio, POS: chromosome position.

## Discussion

We describe a GWAS of ARDS among patients that were mainly affected by severe infections, revealing a genome-wide common genetic variant associated with ARDS risk that has not been reported in previous studies, as well as eight additional suggestively associated loci. Although none of the signals were validated in two additional studies, all nine signals satisfied the stringent internal replication criteria.

The 5q13.3 locus reaching genome-wide significance has been previously associated with increased cholesterol levels [Open targets,^20^]. The risk allele of the highest phenotypic impact variant at this locus, frequent in Europeans, colocalised with increased expression of *ANKDD1B* in tibial artery, while our gene-based rare exonic variant analysis further supported the role of *HMGCR* in ARDS. *ANKDD1B* encodes the Ankyrin Repeat And Death Domain Containing 1B, involved in signal transduction and protein binding, while *HMGCR* encodes the 3-Hydroxy-3-Methylglutaryl-CoA (HMG-CoA) reductase, the rate-limiting enzyme for cholesterol synthesis. Genetic variation in *HMGCR* has been associated with individual responses to statins (HMG-CoA reductase inhibitors) treatment,^20^ and a recent Mendelian randomisation study using genetic instruments has suggested a potential causal relationship between *HMGCR* inhibition and reduced risk of COVID-19 hospitalisation.^21^ However, the use of statins for ARDS remains a topic of ongoing debate. While its use has been supported by several studies in animal and cellular models that reported simvastatin as a protective treatment from lung injury by maintaining the alveolar-capillary barrier integrity and reduce inflammation,^22^^,^^23^ a number of clinical trials have shown no improvement in sepsis or ARDS outcomes.^24^, ^25^, ^26^ Nevertheless, recent studies support that ARDS patients with a hyperinflammatory phenotype^27^ and elevated baseline IL-18 levels^28^ could have a survival benefit from simvastatin treatment, emphasising the importance of clinical characterisation in assessing treatment response. Additionally, a recent multicentre trial study suggested that ARDS patients with low levels of cholesterol had lower mortality when simvastatin was used, encouraging future studies to evaluate this drug in this patient subgroup.^29^ In this context, a computational analysis of omics data in ARDS also supported the cholesterol metabolism dysregulation as one of the main landmarks underlying ARDS pathobiology.^30^ Collectively, these findings highlight the need for further investigation into the role of lipids and potential therapeutic benefits of statins in ARDS.

The 9q22.32 variant associated with ARDS risk, with a high predicted phenotypic impact, was implicated in decreased *FBP1* expression in oesophagus mucosa and pituitary. *FBP1* encodes the fructose-bisphosphatase 1, which is a regulator of appetite and adiposity and whose deficiency is associated with hypoglycaemia.^31^^,^^32^ *FBP1* has been found to be dysregulated under some viral infections, including SARS-CoV-2 infections.^33^^,^^34^ Moreover, Bhargava and colleagues reported that *FBP1* was differentially expressed in bronchoalveolar lavage fluid from ARDS survivors when compared with non-survivors.^35^

We acknowledge some strengths and limitations of this study. Among the strengths of this study, we aimed to address the heterogeneity of the syndrome by comparing ARDS cases with at-risk controls (mainly with severe infections) who did not develop ARDS. This allowed us to identify robust and concordant associations across studies from three different countries, involving different health care systems. Furthermore, we provided further analyses, including gene expression assessment and a rare variant collapsing approach, to support our meta-GWAS findings. A main limitation is the reduced number of patients of non-European genetic ancestry, which limits the ability to evaluate the generalisability of findings to other populations. Additionally, while we used MAMBA to prioritise loci for follow-up analyses, this method is designed for large datasets and may not perform optimally with our limited dataset. In fact, follow-up of the six prioritised variants in two additional studies did not evidence significant findings; therefore, these results should be interpreted with caution. The lack of validation in the GenoSEPSIS study could be due to the limited sample size and subsequent reduced statistical power. Nevertheless, some supporting evidence was observed. Notably, three of the prioritised variants, including the genome-wide significant locus, had the same direction of effect across all five studies, and one variant became slightly more significant when the results were meta-analysed. Independent validation will be necessary to confirm our observations. Another limitation is the UKBB study's reliance on electronic health records for case and control selection, which may result in less thoroughly characterised phenotypes compared to other cohorts, including the absence of APACHE II scores for model adjustment, introducing phenotypic misclassification bias. This may provide an explanation for the non-replication of the variant association at *FLT1* reported in a previous sepsis-associated ARDS GWAS^7^ within the UKBB study. Furthermore, the lack of clinical and demographic characterisation limited the possibility to perform additional sensitivity analyses, including assessing the impact of genetic variants on disease aetiology and severity through subgroup analyses. Finally, although we have provided functional evidence through aggregated experimental data, in vitro analyses are necessary to further investigate the biological implications of the reported findings in relation to ARDS risk.

In summary, our GWAS of ARDS has revealed a previously unreported common variant association with ARDS susceptibility previously linked to cholesterol levels. The reported locus and subsequent rare-variant analysis suggest that *HMGCR* could be important in the pathophysiology of the syndrome. These findings, coupled with the accumulated evidence, underscore the potential of the lipid metabolism pathway as a target for ARDS treatment. Additional independent replication of these signals and further functional evidence is needed to validate our results.

## Contributors

BG-G, ES-P, ET-H, TH-B, JML-S, DC, RG-M, LAR-R, OCL, RJA, MS, and AS performed the analyses. BG-G, ES-P, and CF have accessed and verified the underlying data. AC, RC, MB-C, AC, ET, VEK, LBW, BLY, MS, AS, JV, LVW, and CF participated in data collection. BGG, LVW, and CF supervised the study. BG-G, ES-P, and CF wrote the first draft of the manuscript. All authors revised and approved the final version of the manuscript.

## Data sharing statement

Raw genotype or phenotype data cannot be made available due to restrictions imposed by the ethics approval. Meta-GWAS summary data is currently available for requesters following the instructions provided at https://github.com/genomicsITER/HGinfections, and will be made publicly available through GWAS Catalogue.

## Declaration of interests

DC and BLY are full-time employees of Genentech and hold stock and stock options in Roche. VEK has been supported by the National Heart Lung and Blood Institute (Career Development Grant) and American Thoracic Society (Foundation Research Grant), has received a lecture honorarium from the University of California (San Francisco), and has participated on the following Data Safety Monitoring Boards: Mode of Ventilation During Critical IllnEss (MODE) Trial (Vanderbilt University Medical Center) and ECMO-Free Trial (Vanderbilt University Medical Center). LBW has received consulting fees from Novartis, Healios, Arrowhead and Akebia; is a stockholder in Virtuoso Surgical; has research contracts paid to her institution from Bluejay Diagnostics, Genentech, National Institutes of Health and US Department of Defence; and has served as a Data Safety and Monitoring Board Member for the CHILL trial, and as a Data Monitoring Committee Chair for the SIGNET trial. MS receives funding from Pfizer for a project not related to this research. LVW reports research funding from GlaxoSmithKline, Genentech, Orion Pharma, Wellcome Trust and Medical Research Council; consultancy for Galapagos, Boehringer Ingelheim and GlaxoSmithKline, outside of the submitted work; and honoraria for her work as Associate Editor for European Respiratory Journal and as a Medical Research Council Board member and Deputy Chair. CF has received honoraria in educational events from Fundación Instituto Roche. The other authors declare no competing interests.

## References

[1] Bellani G., Laffey J.G., Pham T. (2016). Epidemiology, patterns of care, and mortality for patients with acute respiratory distress syndrome in intensive care units in 50 countries. JAMA.

[2] ARDS Definition Task Force, Ranieri V.M., Rubenfeld G.D. (2012). Acute respiratory distress syndrome: the Berlin definition. JAMA.

[3] Villar J., Szakmany T., Grasselli G., Camporota L. (2023). Redefining ARDS: a paradigm shift. Crit Care.

[4] Shaw T.D., McAuley D.F., O'Kane C.M. (2019). Emerging drugs for treating the acute respiratory distress syndrome. Expert Opin Emerg Drugs.

[5] Nelson M.R., Tipney H., Painter J.L. (2015). The support of human genetic evidence for approved drug indications. Nat Genet.

[6] Suarez-Pajes E., Tosco-Herrera E., Ramirez-Falcon M. (2023). Genetic determinants of the acute respiratory distress syndrome. J Clin Med.

[7] Guillen-Guio B., Lorenzo-Salazar J.M., Ma S.F. (2020). Sepsis-associated acute respiratory distress syndrome in individuals of European ancestry: a genome-wide association study. Lancet Respir Med.

[8] Willer C.J., Li Y., Abecasis G.R. (2010). METAL: fast and efficient meta-analysis of genomewide association scans. Bioinformatics.

[9] Zhu Z., Zheng Z., Zhang F. (2018). Causal associations between risk factors and common diseases inferred from GWAS summary data. Nat Commun.

[10] McGuire D., Jiang Y., Liu M. (2021). Model-based assessment of replicability for genome-wide association meta-analysis. Nat Commun.

[11] Du M., Garcia J.G.N., Christie J.D. (2021). Integrative omics provide biological and clinical insights into acute respiratory distress syndrome. Intensive Care Med.

[12] Wakefield J. (2009). Bayes factors for genome-wide association studies: comparison with P -values. Genet Epidemiol.

[13] Ghoussaini M., Mountjoy E., Carmona M. (2021). Open targets genetics: systematic identification of trait-associated genes using large-scale genetics and functional genomics. Nucleic Acids Res.

[14] GTEx Consortium (2013). The genotype-tissue expression (GTEx) project. Nat Genet.

[15] Giambartolomei C., Vukcevic D., Schadt E.E. (2014). Bayesian test for colocalisation between pairs of genetic association studies using summary statistics. PLoS Genet.

[16] Lee S., Emond M.J., Bamshad M.J. (2012). Optimal unified approach for rare-variant association testing with application to small-sample case-control whole-exome sequencing studies. Am J Hum Genet.

[17] EPACTS website EPACTS: efficient and parallelizable association container toolbox. http://genome.sph.umich.edu/wiki/EPACTS.

[18] Siew E.D., Ware L.B., Gebretsadik T. (2009). Urine neutrophil gelatinase-associated lipocalin moderately predicts acute kidney injury in critically ill adults. J Am Soc Nephrol.

[19] Martín-Fernández M., Heredia-Rodríguez M., González-Jiménez I. (2022). Hyperoxemia in postsurgical sepsis/septic shock patients is associated with reduced mortality. Crit Care.

[20] Klimentidis Y.C., Arora A., Newell M. (2020). Phenotypic and genetic characterization of lower LDL cholesterol and increased type 2 diabetes risk in the UK biobank. Diabetes.

[21] Huang W., Xiao J., Ji J., Chen L. (2021). Association of lipid-lowering drugs with COVID-19 outcomes from a Mendelian randomization study. Elife.

[22] Terblanche M., Almog Y., Rosenson R.S., Smith T.S., Hackam D.G. (2006). Statins: panacea for sepsis?. Lancet Infect Dis.

[23] Singla S., Jacobson J.R. (2012). Statins as a novel therapeutic strategy in acute lung injury. Pulm Circ.

[24] Lewis S.R., Pritchard M.W., Thomas C.M., Smith A.F. (2019). Pharmacological agents for adults with acute respiratory distress syndrome. Cochrane Database Syst Rev.

[25] McAuley D.F., Laffey J.G., O'Kane C.M. (2018). Simvastatin to reduce pulmonary dysfunction in patients with acute respiratory distress syndrome: the HARP-2 RCT. Efficacy Mechanism Evaluation.

[26] Hofmaenner D.A., Kleyman A., Press A., Bauer M., Singer M. (2022). The many roles of cholesterol in sepsis: a review. Am J Respir Crit Care Med.

[27] Calfee C.S., Delucchi K.L., Sinha P. (2018). Acute respiratory distress syndrome subphenotypes and differential response to simvastatin: secondary analysis of a randomised controlled trial. Lancet Respir Med.

[28] Boyle A.J., Ferris P., Bradbury I. (2022). Baseline plasma IL-18 May predict simvastatin treatment response in patients with ARDS: a secondary analysis of the HARP-2 randomised clinical trial. Crit Care.

[29] Pienkos S.M., Moore A.R., Guan J. (2023). Effect of total cholesterol and Statin therapy on mortality in ARDS patients: a secondary analysis of the SAILS and HARP-2 trials. Crit Care.

[30] Millar J.E., Clohisey-Hendry S., McMannus M. (2024). The genomic landscape of acute respiratory distress syndrome: a meta-analysis by information content of genome-wide studies of the host response. medRxiv.

[31] Liang X., Liu X., Li W. (2023). A novel variant in the FBP1 gene causes fructose-1,6-bisphosphatase deficiency through increased ubiquitination. Arch Biochem Biophys.

[32] Bijarnia-Mahay S., Bhatia S., Arora V., Adam M.P., Feldman J., Mirzaa G.M. (1993). GeneReviews®.

[33] Zhang Z., Wang T., Liu F. (2021). The proteomic characteristics of airway mucus from critical ill COVID-19 patients. Life Sci.

[34] Sengupta I., Mondal P., Sengupta A. (2022). Epigenetic regulation of Fructose-1,6-bisphosphatase 1 by host transcription factor speckled 110 kDa during hepatitis B virus infection. FEBS J.

[35] Bhargava M., Viken K., Wang Q. (2017). Bronchoalveolar lavage fluid protein expression in acute respiratory distress syndrome provides insights into pathways activated in subjects with different outcomes. Sci Rep.

