# Supplementary material for: Genome-wide association study of susceptibility to acute respiratory distress syndrome
Source: eBioMedicine. 2025 Sep 30;120:105951. doi: 10.1016/j.ebiom.2025.105951 (PMC12516505; doi:10.1016/j.ebiom.2025.105951)
Supplement: Supplementary Figures and Tables [file mmc1.pdf]

Appendix

Genome-wide association study of susceptibility to acute respiratory distress syndrome

Beatriz Guillen-Guio, Eva Suarez-Pajes, Eva Tosco-Herrera, Tamara Hernandez-Beeftink, Jose M. Lorenzo-Salazar, Diana Chang, Rafaela González-Montelongo, Luis A. Rubio-Rodríguez, Olivia C. Leavy, Richard J. Allen, Almudena Corrales, Raquel Cruz, Miguel Bardají-Carrillo, Angel Carracedo, Eduardo Tamayo, V. Eric Kerchberger, Lorraine B. Ware, Brian L. Yaspan, Markus Scholz, André Scherag, Jesús Villar, Louise V. Wain*, Carlos Flores*

*Equal contribution as senior authors

Supplementary Methods

Sample description

We performed a GWAS meta-analysis on 5,115 critically-ill patients from three independent studies (**Figure 1**): the GENetics of SEPsis-induced ARDS Network (GEN-SEP), The Critical Care Trials Group of the German Sepsis Competence Network (SepNet), and UK Biobank (UKBB). Cases were defined as critical patients who developed ARDS, mainly associated with severe infections, while the majority of controls comprised critically ill patients who did not develop ARDS. The study comprised a total of 716 ARDS cases and 4,399 at-risk controls, including 805 patients from the GEN-SEP cohort (304 cases and 501 at-risk controls), 740 from SepNet (91 cases and 649 at-risk controls), and 3,570 from UKBB (321 cases and 3,249 at-risk controls).

In GEN-SEP and SepNet, patients with a diagnosis of sepsis (Singer et al., 2016) admitted to the Intensive Care Units (ICUs) were followed for ARDS development according to Berlin definition criteria (Force et al., 2012). GEN-SEP is a multicentre, observational study conducted in a Spanish network of ICUs comprising adult patients with all-cause sepsis of European ancestry recruited in two periods (in 2002-2015 and in 2016-2019) (Hernandez-Beeftink et al., 2022). The GEN-SEP study was approved by the Ethics Committee from the Hospital Universitario de Canarias (CHUNSC_2018-16 and CHUNSC_2021-40). SepNet is integrated by the VISEP (recruitment in 2003-2005, registration number: NCT00135473) and MAXSEP (recruitment in 2007-2010, registration number: NCT00534287) randomised controlled trials and includes European ancestry patients with all-cause sepsis from German hospitals (Scherag et al., 2016).

For the UKBB cohort, ARDS cases were obtained from UK Biobank based on the International Classification of Diseases 10th Revision (ICD-10) diagnosis code J80 ("Adult respiratory distress syndrome"). Of these, 75.4% of ARDS cases were associated with severe infections (45.17% with pneumonia, 21.18% with pneumonia and septicaemia, and 9.03% with septicaemia). The remaining 24.6% ARDS cases were associated with infections due to complications of surgical and medical care (2.18%), pancreatitis (1.56%), major injury (1.56%), inhalation of harmful substances (0.93%), drug poisoning (0.62%), and unspecified "other causes" (17.76%). The inclusion of non-septicaemia patients aims to enhance the statistical power of the study. The list of ICD-10 codes used to determine the ARDS development causes is provided in **Table S1**. More than 92% of individuals were of European ancestry (**Table S2**). Controls in the UKBB study comprised critically ill patients matched for ARDS development cause (i.e., 75.4% pneumonia and/or septicaemia, 24.6% other causes), sex, age, and ancestry. UK Biobank has approval from the North West Multi-centre Research Ethics Committee (MREC).

Genotyping, imputation and association studies

In GEN-SEP, single nucleotide polymorphism (SNP) genotyping was performed using the Axiom Genome-Wide Human CEU 1 array (Thermo Fisher Scientific) in the National Genotyping Center (CeGen), Universidad de Santiago de Compostela Node, Spain. Variant calling was done with AffyPipe v.2.10.0, following the manufacturer recommendations. Genotyping quality control was conducted using PLINK v.1.9 and R v.3.6.0 in order to exclude patients with missing clinical information, call rates (CR) <95%, sex discordance, high kinship degree (PIHAT>0.2), or heterozygosity outliers. Those variants with CR<95%, minor allele frequency (MAF) <1%, or that strongly deviated from the Hardy Weinberg equilibrium (HWE) expectations ($p<1.0\times10^{-6}$) were excluded from the analyses. We performed a principal component (PC) analysis (PCA) based on >114,000 independent SNPs to identify and exclude ancestry outliers. Variant imputation was performed with the Michigan Imputation Server using the Haplotype Reference Consortium (HRC) panel v.1.1 (Loh et al., 2016). For variants with

MAF>1% and high imputation quality (Rsq>0.3), we performed genetic association testing with EPACTS v.3.2.6 (EPACTS website) using a logistic regression model. We adjusted for sex, age, and the Acute Physiology and Chronic Health Evaluation II (APACHE II) score as described in our prior study (Guillen-Guio et al., 2020). No PCs were included in the model due to the absence of substructure in this cohort. For the X chromosome, we followed the quality controls described above, separately for males and females. In addition, a SNP filtering was performed based on the HWE deviations calculated from females, and results for males and females were then meta-analysed using METASOFT (Han and Eskin, 2011).

In SepNet, SNP genotyping was obtained with the HumanOmniExpressExome array (Illumina, Inc.). Those patients without discordance between the declared and genetic sex, CR<98%, implausible heterozygosity (<20% and >26%) or that were detected as genetic outliers based on PCA were excluded. Variants with a CR≤95%, MAF<1%, or strongly deviating from HWE (p<1.0x10^{-6}) were also removed from the analyses. Variant imputation was obtained in prephased data from SHAPEIT v.2.r790 (Delaneau et al., 2012) using IMPUTE2 v.2.3.0 (Howie et al., 2009) and the 1000 Genomes Project data (phase1, v3) as a reference panel (The 1000 Genomes Project Consortium, 2015). Logistic regression was used for genetic association testing with SNPTEST v.2.5 (Marchini and Howie, 2010) for variants with MAF>1%, HWE p-value>1.0x10^{-10}, and high imputation quality (INFO>0.8). The first three genetic PCs, sex, age, and APACHE II were included in the model as covariates. Only autosomes were evaluated in this study.

In UKBB, imputed genetic data and phenotypes were obtained through UK Biobank Application 648 (Bycroft et al., 2018). Genotyping was done using the UK BiLEVE and Applied Biosystems UK Biobank Axiom Arrays (Thermo Fisher Scientific). Individuals were removed if they had a final individual CR<95%, discordance between recorded and genetically inferred sex, were related (Kinship≥0.15), or if they had an extreme PC-adjusted heterozygosity rate. Ethnic groups were defined through K-means clustering using the first two PCs, as previously described (Shrine et al., 2019). Only variants included on both the UK BiLEVE and UK Biobank Axiom arrays, and satisfying a HWE p>1.0x10^{-6}, CR≥90%, and MAF≥ 0.0001 were considered. HRC served as the primary reference panel for variant imputation (Bycroft et al., 2018). Additionally, imputation was conducted with the combined UK10K and 1000 Genomes phase 3 reference panels, integrating the results with those from HRC. Quality controls were consistently applied to chromosome X. HWE deviation analysis was conducted exclusively on females on the sex-specific region of the X chromosome. Logistic regression analyses were done using PLINK v2.00 (Chang et al., 2015) and models were adjusted for the first ten genetic PCs, sex, and age. Only variants with MAF>1% and an imputation quality score ≥0.3 were considered in the analyses.

Statical power calculation

Statistical power was estimated using Quanto, assuming an additive inheritance model for a GWAS of 716 cases and 4,399 controls. The study had >74% statistical power to detect a significant association at p=5x10^{-8} for variants with MAF of at least 0.21.

Bayesian fine-mapping

We performed a Bayesian fine mapping on the association results around 1 Mb of each sentinel variant (significantly or suggestively associated), considering all with a r^2>0.1 with the sentinel variant. The posterior probabilities (PPs) were calculated from the approximate Bayes factors (ABFs) using the formula proposed by Wakefield (Wakefield, 2009):

$$ABF = \frac{1}{\sqrt{1-\frac{W}{V+w}}} exp(-\frac{Z^2 W}{2V+W})$$

Where, for each variant, V is the variance of the effect size, W is the Wakefield prior of 0.4, equivalent to a 95% confidence, and Z is the Z-score.

The approximate posterior probability for each variant was set as its ABF divided by the sum of the ABFs of all variants in the locus. Variants were sorted into descending order of their computed posterior probability and then cumulatively summed until the credible set threshold was exceeded (>0.95).

In-silico functional assessment: colocalisation analysis

To evaluate whether the causal variants driving genetic associations with ARDS also regulate gene expression, we performed colocalisation analysis. We accessed gene expression data from GTEx (Genotype-Tissue Expression) Release v.8 and selected relevant genes whose lead variant was a significant expression quantitative trait locus (eQTL) in tissues of interest for the disease (i.e. lung, cultured fibroblasts, whole blood, thyroid, tibial and aorta artery, Epstein-Barr virus-transformed lymphocytes and oesophagus mucosa). The significance threshold was set at $p<6.25x10^{-3}$ after correcting for the eight tissues. Colocalisation was evaluated using the *coloc* R package (Giambartolomei et al., 2014), which assumes a single causal variant per locus and evaluates the following hypotheses: H0: no association with either ARDS susceptibility or gene expression; H1: association with ARDS susceptibility only; H2: association with gene expression only; H3: association with ARDS susceptibility and gene expression, but due to different causal variants; H4: association with ARDS susceptibility and gene expression, driven by the same causal variant. Colocalisation was considered supported when the posterior probability (PP) for H4 was > 70%.

Whole-exome sequencing, bioinformatics processing, and variant annotation

Whole-exome sequencing was available from 260 patients with sepsis-associated ARDS and 537 at-risk controls with sepsis from the GEN-SEP study.

Libraries were prepared using the DNA Prep with Enrichment kit (Illumina Inc.) or SureSelect XT HS2 DNA (Agilent Technologies Inc.) as detailed elsewhere (Diaz-de Usera et al., 2020). Library sizes and concentration were obtained with a TapeStation 4200 (Agilent Technologies Inc.) and the Qubit dsDNA HS Assay (Thermo Fisher Scientific). Sequencing was conducted on Illumina NextSeq550, HiSeq4000, or NovaSeq6000 using 75 or 100 base paired-end reads (depending on the instrument) to an average of 100X depth and including 1% of PhiX control V3 (Illumina Inc.). Sequencing was done at Instituto Tecnológico y de Energías Renovables (ITER, Santa Cruz de Tenerife, Spain).

Pre-processing of sequencing data was conducted by bcl2fastq v.2.18 for demultiplexing and BWA-MEM v.0.7.15 (Li and Durbin, 2009) to align reads to the GRCh37/hg19 reference. Resulting BAM files were assessed using SAMtools v.1.3 (Danecek et al., 2021) and Picard v.2.10.10 (Picard website). Calling of small germline variants (SNPs and indels of up to 50 bp) was performed using GATK HaplotypeCaller v.3.8 (Poplin et al., 2018) with a padding of 100 bp around the capture targets and following the GATK Best Practices workflow recommendations. Variants were filtered using BCFtools v.1.16 (Danecek et al., 2021) based on "PASS" filter, missingness (FMISS) ≤0.05, genotype quality (GQ) ≥20, and depth of coverage (DP) ≥10. Functional annotation of variants was performed on the resulting filtered callsets located on the GWAS-prioritised genes. Variants were annotated for population allele frequency, variant type, protein function, and pathogenic potential with Ensembl

Variant Effect Predictor (VEP) v.105 and ANNOVAR v.07.06.20 (Wang et al., 2010) based on different databases. Analyses were conducted at the Teide-HPC supercomputing facility (http://teidehpc.iter.es/en).

Rare exonic variant association testing

To assess the association of gene-based rare exonic variation with SKAT-O, we selected the genes that were closest to the prioritised variant and those with the highest functional relevance in the colocalisation (H3 or H4>70%). We performed these tests including only the variants with MAF≤0.01 (i.e., with MAF≤0.01 in The 1000 Genomes Project Phase 3 or in gnomAD v.2.1.1, or that were absent from these sources but present in the GEN-SEP patients) or only the variants that had MAF≤0.01 and considered of likely high biological impact based on scaled CADD score > Mutation Significance Cutoff (MSC) or with CADD >15 in absence of MSC information for the given gene.

Validation cohorts

The Validating Acute Lung Injury biomarkers for Diagnosis (VALID) study comprised 1,106 patients with sepsis (Siew et al., 2009). The criterion for classifying a patient as a case (n=385) was that the patient had Berlin definition of ARDS on two consecutive days and was on mechanical ventilation for at least one day. At-risk controls (n=721) were those ICU patients with sepsis participating in the trial without ARDS at enrolment or for the next three days of follow-up. SNP genotyping was done with a custom Global Screening Array (Illumina Inc.) and variants were imputed using a reference panel consisting of Genentech's whole-genome sequencing data plus The 1000 Genomes Project dataset (all at a depth of 30X) (Sudmant et al., 2015). Variant imputation was done using BEAGLE v.5.1 (Browning et al., 2018) and association testing was carried out using logistic regression models in PLINK v.1.9 (Purcell et al., 2007), correcting for genetically inferred sex, age, and first five PCs. All individuals included in the analysis had ≥70% European genetic ancestry according to ADMIXTURE v.1.3 (Alexander et al., 2009).

The Genomic Study of Sepsis (GenoSEPSIS) cohort consisted of 722 Spanish ICU patients recruited in the Hospital Clinico Universitario de Valladolid (Spain) (Martin-Fernandez et al., 2022). This study included 45 ARDS cases according to Berlin definition criteria (Force et al., 2012) and 677 at-risk controls, mostly with septic shock. SNP genotyping was done with the Spain Biobank Array (Thermo Fisher Scientific) in the National Genotyping Center (CeGen), Universidad de Santiago de Compostela Node, Spain. Quality control, filtering, imputation, and association testing were conducted following the same procedures as in the GEN-SEP study and adjusting for the first four PCs and sex.

Both studies were approved by the relevant Research Ethics Committees (Vanderbilt IRB #051065 for VALID and PI 20-2070 for GenoSEPSIS).

Supplementary References

Alexander, D. H., Novembre, J. & Lange, K. 2009. Fast model-based estimation of ancestry in unrelated individuals. *Genome Res,* 19(9)**,** pp 1655-64.

Browning, B. L., Zhou, Y. & Browning, S. R. 2018. A One-Penny Imputed Genome from Next-Generation Reference Panels. *Am J Hum Genet,* 103(3)**,** pp 338-348.

Bycroft, C., Freeman, C., Petkova, D., Band, G., Elliott, L. T., Sharp, K., Motyer, A., Vukcevic, D., Delaneau, O., O'Connell, J., Cortes, A., Welsh, S., Young, A., Effingham, M., McVean, G., Leslie, S., Allen, N., Donnelly, P. & Marchini, J. 2018. The UK Biobank resource with deep phenotyping and genomic data. *Nature,* 562(7726)**,** pp 203-209.

Chang, C. C., Chow, C. C., Tellier, L. C., Vattikuti, S., Purcell, S. M. & Lee, J. J. 2015. Second-generation PLINK: rising to the challenge of larger and richer datasets. *Gigascience,* 4(7.

Danecek, P., Bonfield, J. K., Liddle, J., Marshall, J., Ohan, V., Pollard, M. O., Whitwham, A., Keane, T., McCarthy, S. A., Davies, R. M. & Li, H. 2021. Twelve years of SAMtools and BCFtools. *Gigascience,* 10(2)**,** pp.

Delaneau, O., Marchini, J. & Zagury, J. F. 2012. A linear complexity phasing method for thousands of genomes. *Nat Methods,* 9(2)**,** pp 179-81.

Diaz-de Usera, A., Lorenzo-Salazar, J. M., Rubio-Rodriguez, L. A., Munoz-Barrera, A., Guillen-Guio, B., Marcelino-Rodriguez, I., Garcia-Olivares, V., Mendoza-Alvarez, A., Corrales, A., Inigo-Campos, A., Gonzalez-Montelongo, R. & Flores, C. 2020. Evaluation of Whole-Exome Enrichment Solutions: Lessons from the High-End of the Short-Read Sequencing Scale. *J Clin Med,* 9(11)**,** pp.

EPACTS website. *EPACTS: Efficient and Parallelizable Association Container Toolbox* [Online]. Available: http://genome.sph.umich.edu/wiki/EPACTS.

Force, A. D. T., Ranieri, V. M., Rubenfeld, G. D., Thompson, B. T., Ferguson, N. D., Caldwell, E., Fan, E., Camporota, L. & Slutsky, A. S. 2012. Acute respiratory distress syndrome: the Berlin Definition. *JAMA,* 307(23)**,** pp 2526-33.

Giambartolomei, C., Vukcevic, D., Schadt, E. E., Franke, L., Hingorani, A. D., Wallace, C., & Plagnol, V. (2014). Bayesian Test for Colocalisation between Pairs of Genetic Association Studies Using Summary Statistics. *PLoS Genetics*, 10(5), e1004383.

Han, B. & Eskin, E. 2011. Random-effects model aimed at discovering associations in meta-analysis of genome-wide association studies. *Am J Hum Genet,* 88(5)**,** pp 586-98.

Hernandez-Beeftink, T., Guillen-Guio, B., Lorenzo-Salazar, J. M., Corrales, A., Suarez-Pajes, E., Feng, R., Rubio-Rodriguez, L. A., Paynton, M. L., Cruz, R., Garcia-Laorden, M. I., Prieto-Gonzalez, M., Rodriguez-Perez, A., Carriedo, D., Blanco, J., Ambros, A., Gonzalez-Higueras, E., Espinosa, E., Muriel, A., Tamayo, E., Martin, M. M., Lorente, L., Dominguez, D., de Lorenzo, A. G., Giannini, H. M., Reilly, J. P., Jones, T. K., Anon, J. M., Soro, M., Carracedo, A., Wain, L. V., Meyer, N. J., Villar, J., Flores, C. & Genetics of Sepsis, N. 2022. A genome-wide association study of survival in patients with sepsis. *Crit Care,* 26(1)**,** pp 341.

Howie, B. N., Donnelly, P. & Marchini, J. 2009. A flexible and accurate genotype imputation method for the next generation of genome-wide association studies. *PLoS Genet,* 5(6)**,** pp e1000529.

Li, H. & Durbin, R. 2009. Fast and accurate short read alignment with Burrows-Wheeler transform. *Bioinformatics,* 25(14)**,** pp 1754-60.

Loh, P. R., Danecek, P., Palamara, P. F., Fuchsberger, C., Y, A. R., H, K. F., Schoenherr, S., Forer, L., McCarthy, S., Abecasis, G. R., Durbin, R. & A, L. P. 2016. Reference-based phasing using the Haplotype Reference Consortium panel. *Nat Genet,* 48(11)**,** pp 1443-1448.

Marchini, J. & Howie, B. 2010. Genotype imputation for genome-wide association studies. *Nat Rev Genet,* 11(7)**,** pp 499-511.

Martin-Fernandez, M., Heredia-Rodriguez, M., Gonzalez-Jimenez, I., Lorenzo-Lopez, M., Gomez-Pesquera, E., Poves-Alvarez, R., Alvarez, F. J., Jorge-Monjas, P., Beltran-DeHeredia, J., Gutierrez-Abejon, E., Herrera-Gomez, F., Guzzo, G., Gomez-Sanchez, E., Tamayo-Velasco, A., Aller, R., Pelosi, P., Villar, J. & Tamayo, E. 2022. Hyperoxemia in postsurgical sepsis/septic shock patients is associated with reduced mortality. *Crit Care,* 26(1)**,** pp 4.

Picard website. *"Picard Tools." Broad Institute, GitHub repository. http://broadinstitute.github.io/picard/* [Online].

Poplin, R., Ruano-Rubio, V., DePristo, M. A., Fennell, T. J., Carneiro, M. O., Auwera, G. A. V. d., Kling, D. E., Gauthier, L. D., Levy-Moonshine, A., Roazen, D., Shakir, K., Thibault, J., Chandran, S., Whelan, C., Lek, M., Gabriel, S., Daly, M. J., Neale, B., MacArthur, D. G. & Banks, E. 2018. Scaling accurate genetic variant discovery to tens of thousands of samples. *bioRxiv,* 201178.

Purcell, S., Neale, B., Todd-Brown, K., Thomas, L., Ferreira, M. A., Bender, D., Maller, J., Sklar, P., de Bakker, P. I., Daly, M. J. & Sham, P. C. 2007. PLINK: a tool set for whole-genome association and population-based linkage analyses. *Am J Hum Genet,* 81(3)**,** pp 559-75.

Scherag, A., Schoneweck, F., Kesselmeier, M., Taudien, S., Platzer, M., Felder, M., Sponholz, C., Rautanen, A., Hill, A. V. S., Hinds, C. J., Hossain, H., Suttorp, N., Kurzai, O., Slevogt, H., Giamarellos-Bourboulis, E. J., Armaganidis, A., Trips, E., Scholz, M. & Brunkhorst, F. M. 2016. Genetic Factors of the Disease Course after Sepsis: A Genome-Wide Study for 28Day Mortality. *EBioMedicine,* 12(239-246.

Shrine, N., Guyatt, A. L., Erzurumluoglu, A. M., Jackson, V. E., Hobbs, B. D., Melbourne, C. A., Batini, C., Fawcett, K. A., Song, K., Sakornsakolpat, P., Li, X., Boxall, R., Reeve, N. F., Obeidat, M., Zhao, J. H., Wielscher, M., Weiss, S., Kentistou, K. A., Cook, J. P., Sun, B. B., Zhou, J., Hui, J., Karrasch, S., Imboden, M., Harris, S. E., Marten, J., Enroth, S., Kerr, S. M., Surakka, I., Vitart, V., Lehtimaki, T., Allen, R. J., Bakke, P. S., Beaty, T. H., Bleecker, E. R., Bosse, Y., Brandsma, C. A., Chen, Z., Crapo, J. D., Danesh, J., DeMeo, D. L., Dudbridge, F., Ewert, R., Gieger, C., Gulsvik, A., Hansell, A. L., Hao, K., Hoffman, J. D., Hokanson, J. E., Homuth, G., Joshi, P. K., Joubert, P., Langenberg, C., Li, X., Li, L., Lin, K., Lind, L., Locantore, N., Luan, J., Mahajan, A., Maranville, J. C., Murray, A., Nickle, D. C., Packer, R., Parker, M. M., Paynton, M. L., Porteous, D. J., Prokopenko, D., Qiao, D., Rawal, R., Runz, H., Sayers, I., Sin, D. D., Smith, B. H., Soler Artigas, M., Sparrow, D., Tal-Singer, R., Timmers, P., Van den Berge, M., Whittaker, J. C., Woodruff, P. G., Yerges-Armstrong, L. M., Troyanskaya, O. G., Raitakari, O. T., Kahonen, M., Polasek, O., Gyllensten, U., Rudan, I., Deary, I. J., Probst-Hensch, N. M., Schulz, H., James, A. L., Wilson, J. F., Stubbe, B., Zeggini, E., Jarvelin, M. R., Wareham, N., Silverman, E. K., Hayward, C., Morris, A. P., et al. 2019. New genetic signals for lung function highlight pathways and chronic obstructive pulmonary disease associations across multiple ancestries. *Nat Genet,* 51(3)**,** pp 481-493.

Siew, E. D., Ware, L. B., Gebretsadik, T., Shintani, A., Moons, K. G., Wickersham, N., Bossert, F. & Ikizler, T. A. 2009. Urine neutrophil gelatinase-associated lipocalin moderately predicts acute kidney injury in critically ill adults. *J Am Soc Nephrol,* 20(8)**,** pp 1823-32.

Singer, M., Deutschman, C. S., Seymour, C. W., Shankar-Hari, M., Annane, D., Bauer, M., Bellomo, R., Bernard, G. R., Chiche, J. D., Coopersmith, C. M., Hotchkiss, R. S., Levy, M. M., Marshall, J. C., Martin, G. S., Opal, S. M., Rubenfeld, G. D., van der Poll, T., Vincent, J. L. & Angus, D. C. 2016. The Third International Consensus Definitions for Sepsis and Septic Shock (Sepsis-3). *JAMA,* 315(8)**,** pp 801-10.

Sudmant, P. H., Rausch, T., Gardner, E. J., Handsaker, R. E., Abyzov, A., Huddleston, J., Zhang, Y., Ye, K., Jun, G., Fritz, M. H., Konkel, M. K., Malhotra, A., Stutz, A. M., Shi, X., Casale, F. P., Chen, J., Hormozdiari, F., Dayama, G., Chen, K., Malig, M., Chaisson, M. J. P., Walter, K., Meiers, S., Kashin, S., Garrison, E., Auton, A., Lam, H. Y. K., Mu, X. J., Alkan, C., Antaki, D., Bae, T., Cerveira, E., Chines, P., Chong, Z., Clarke, L., Dal, E., Ding, L., Emery, S., Fan, X., Gujral, M., Kahveci, F., Kidd, J. M., Kong, Y., Lameijer, E. W., McCarthy, S., Flicek, P., Gibbs, R. A., Marth, G., Mason, C. E., Menelaou, A., Muzny, D. M., Nelson, B. J., Noor, A., Parrish, N. F., Pendleton, M., Quitadamo, A., Raeder, B., Schadt, E. E., Romanovitch, M., Schlattl, A., Sebra, R., Shabalin, A. A., Untergasser, A., Walker, J. A., Wang, M., Yu, F., Zhang, C., Zhang, J., Zheng-Bradley, X., Zhou, W., Zichner, T., Sebat, J., Batzer, M. A., McCarroll, S. A., Genomes Project, C., Mills, R. E., Gerstein, M. B., Bashir, A., Stegle, O.,

Devine, S. E., Lee, C., Eichler, E. E. & Korbel, J. O. 2015. An integrated map of structural variation in 2,504 human genomes. *Nature,* 526(7571)**,** pp 75-81.

Wakefield, J. 2009. Bayes factors for genome-wide association studies: comparison with P-values. *Genet Epidemiol,* 33(1)**,** pp 79-86.

Wang, K., Li, M. & Hakonarson, H. 2010. ANNOVAR: functional annotation of genetic variants from high-throughput sequencing data. *Nucleic Acids Res,* 38(16)**,** pp e164.

Supplementary Figures

Figure S1. Quantile-Quantile (Q-Q) plot. Observed versus expected -log10 *p*-values for the GWAS meta-analysis result (lambda=0.99).

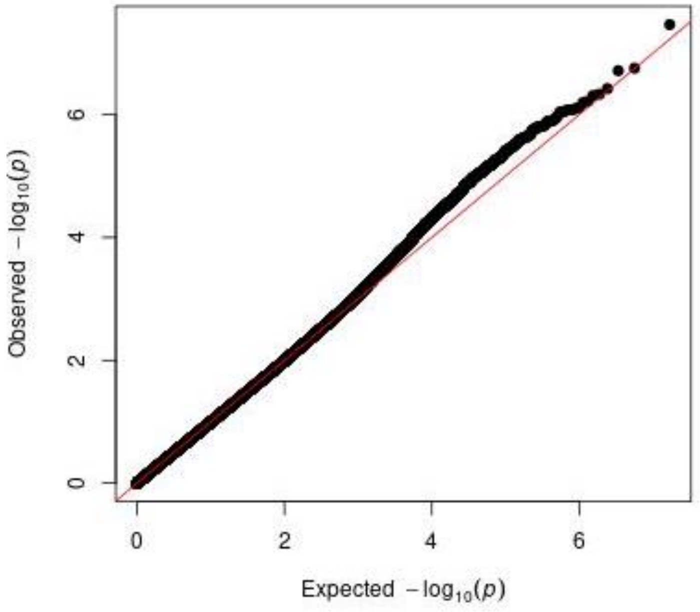

Figure S2. Forest plots of the association results for the nine prioritised variants showing the odds ratios with 95% confidence intervals.

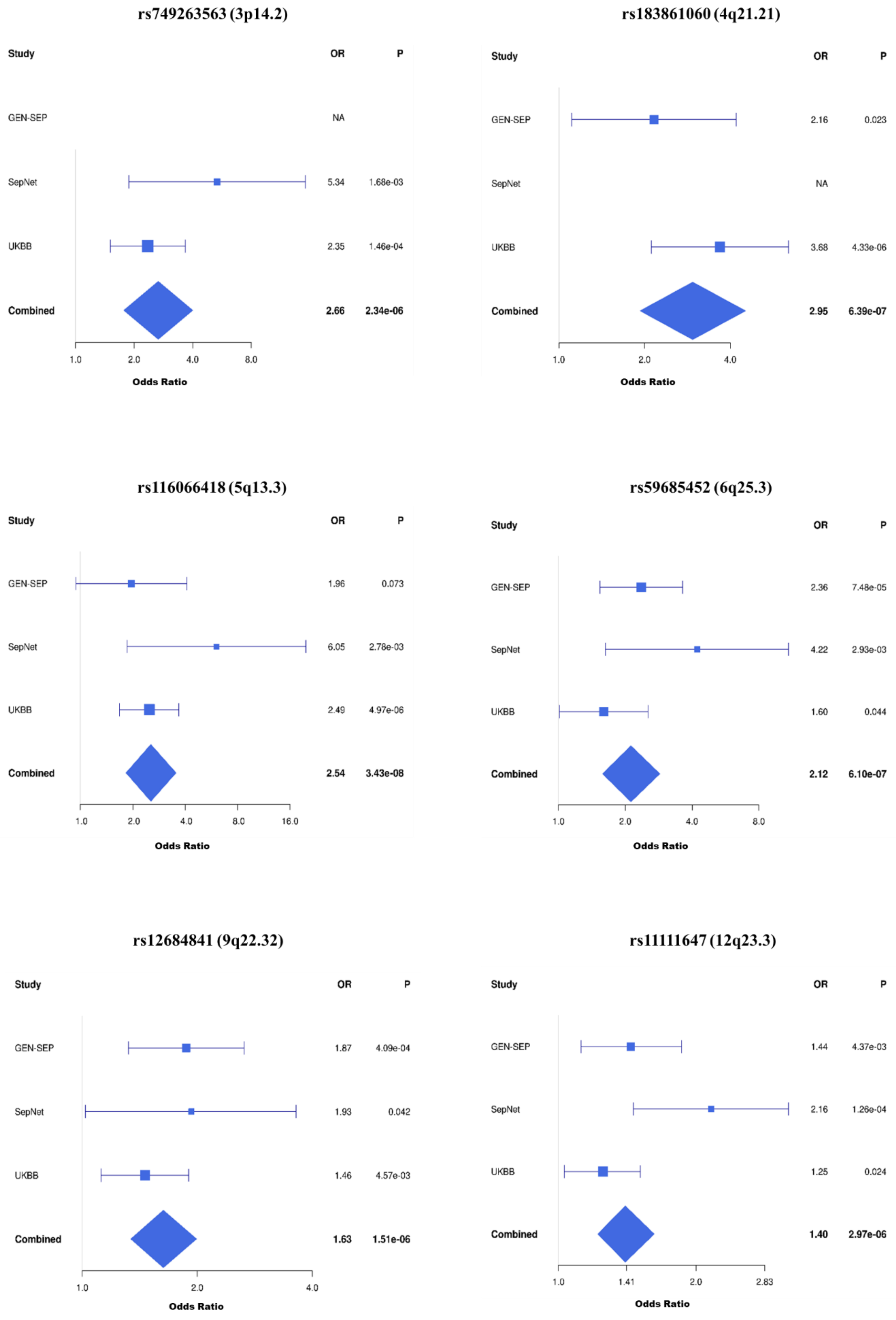

rs118026254 (12q24.22)

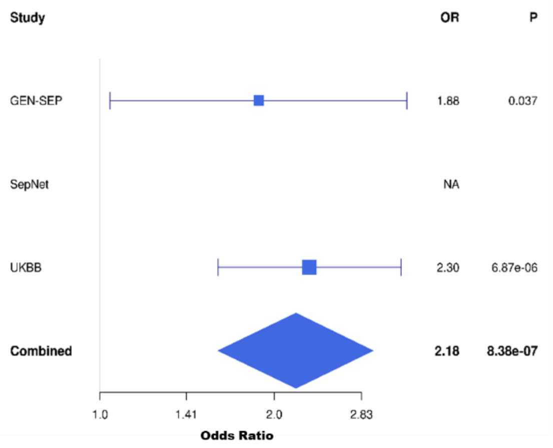

rs8022645 (14q23.3)

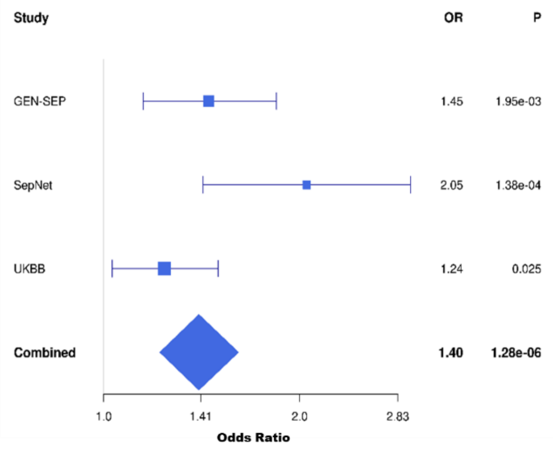

rs4989808 (15q26.1)

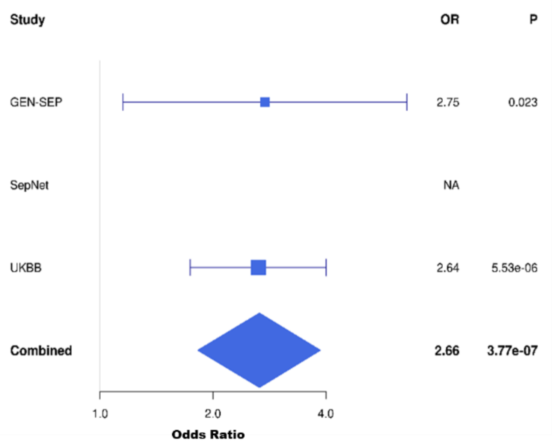

Figure S3. Regional plots of the association results for the prioritised loci. The y-axis shows the transformed *p*-values (-log10[*p*-value]), and the x-axis represents the chromosome positions (GRCh37/hg19). Genome-wide significance threshold (*p*-value=5.0x10^{-8}) is indicated by the horizontal dashed line. Linkage disequilibrium (LD) values (r^2) are based on the European population data from The 1000 Genomes Project and are represented according to the LD colour scheme of the top left legend. The plots were generated with LocusZoom (http://locuszoom.org/).

rs749263563 (3p14.2)

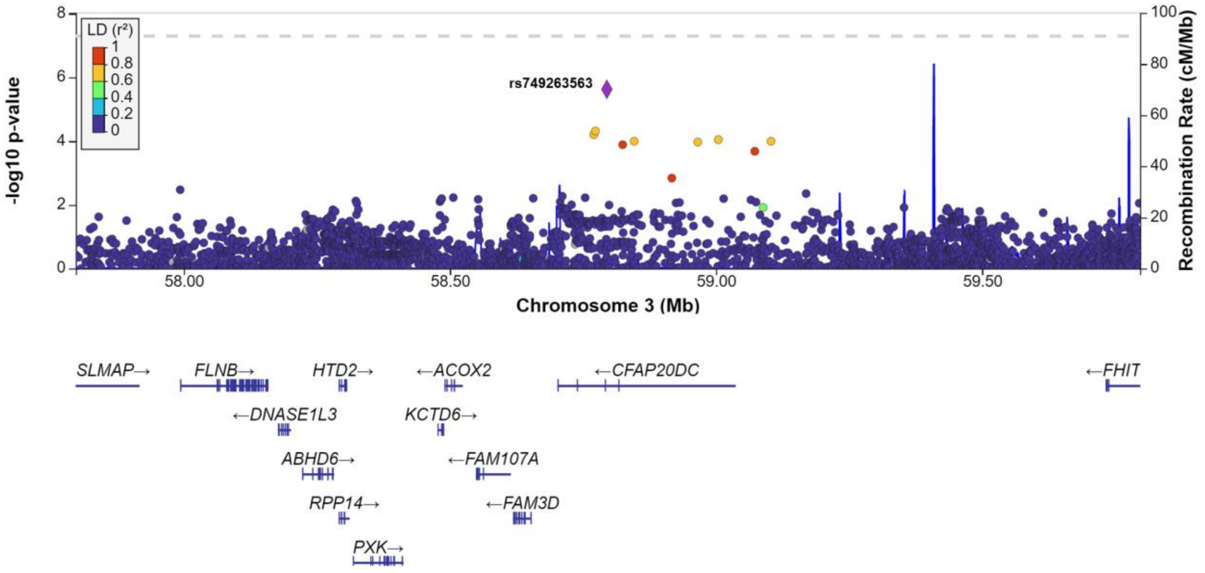

rs183861060 (4q21.21)

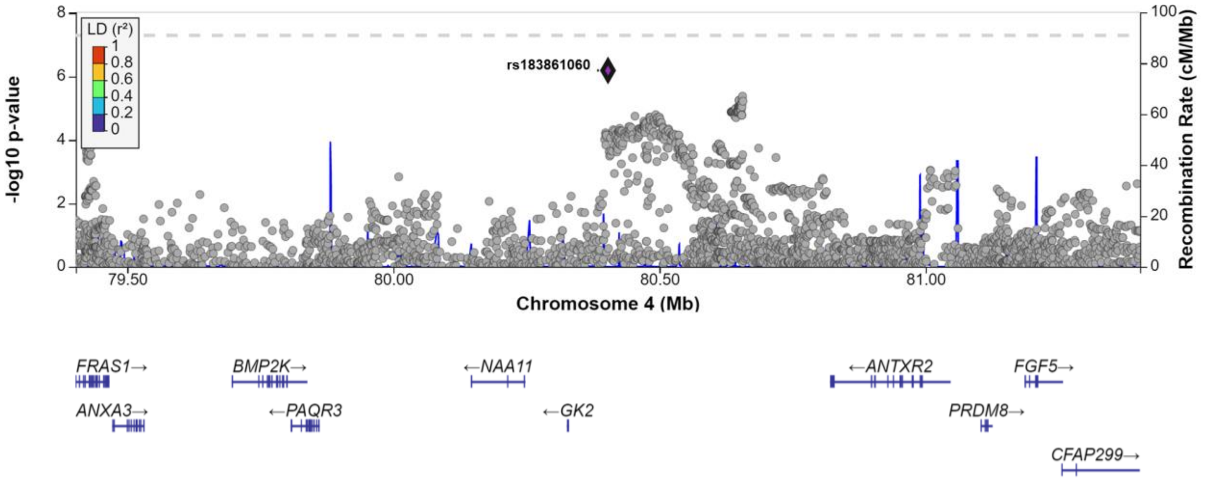

rs116066418 (5q13.3)

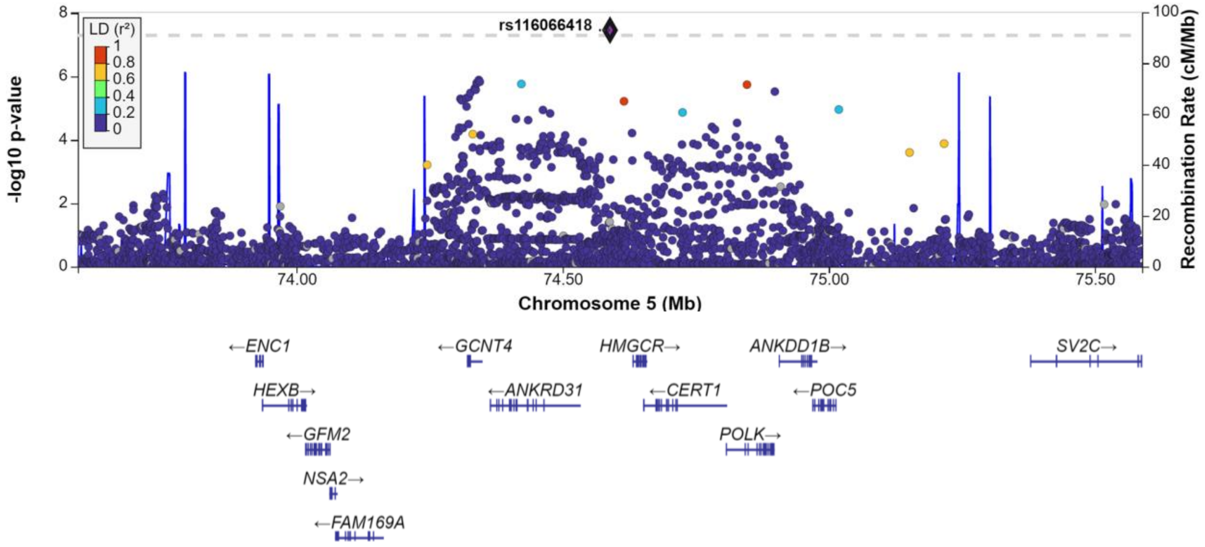

rs59685452 (6q25.3)

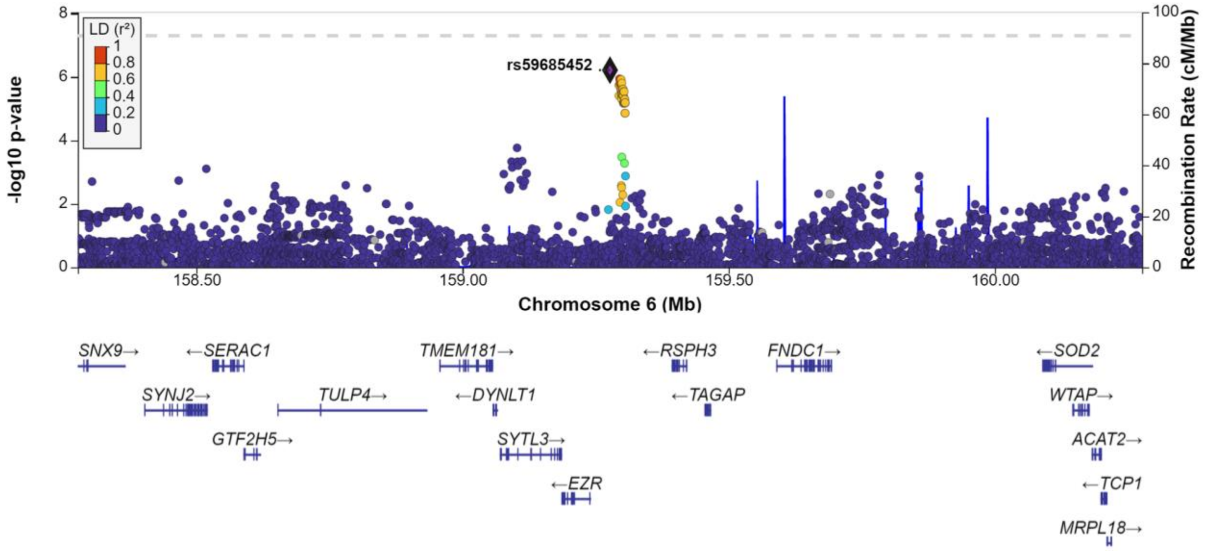

rs12684841 (9q22.32)

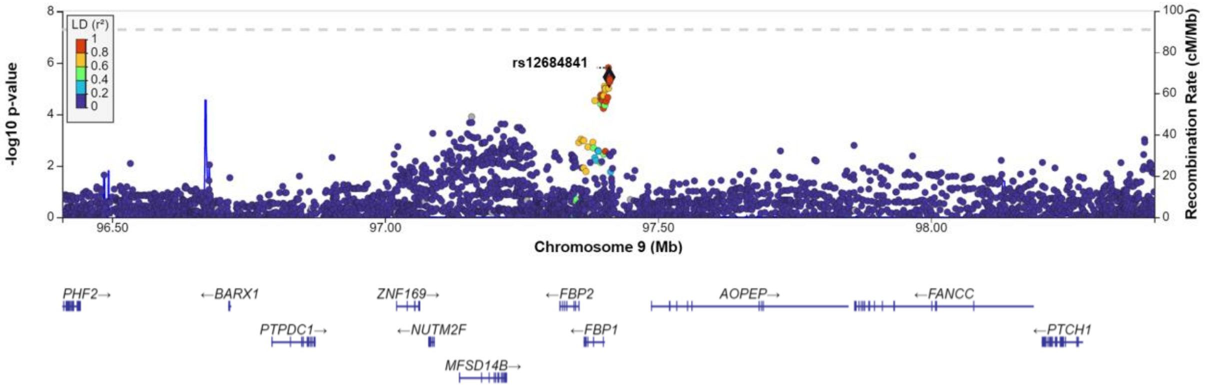

rs11111647 (12q23.3)

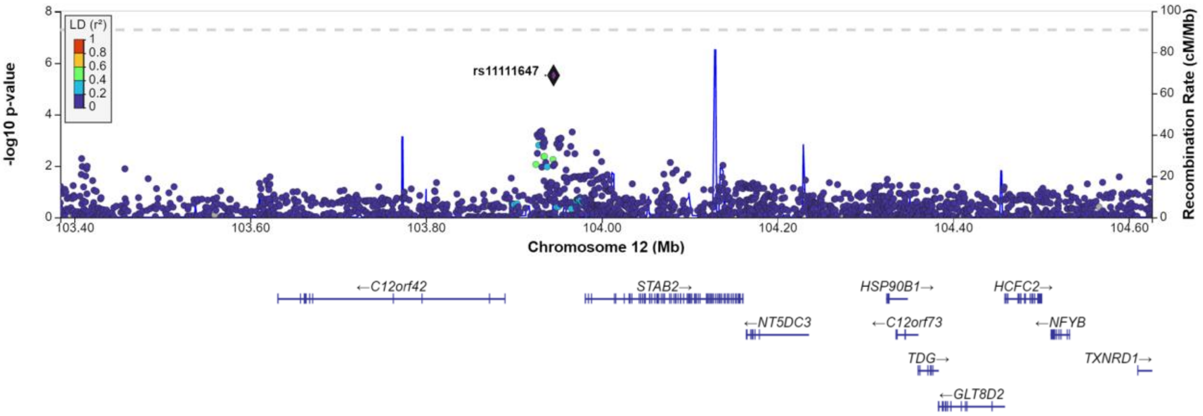

rs118026254 (12q24.22)

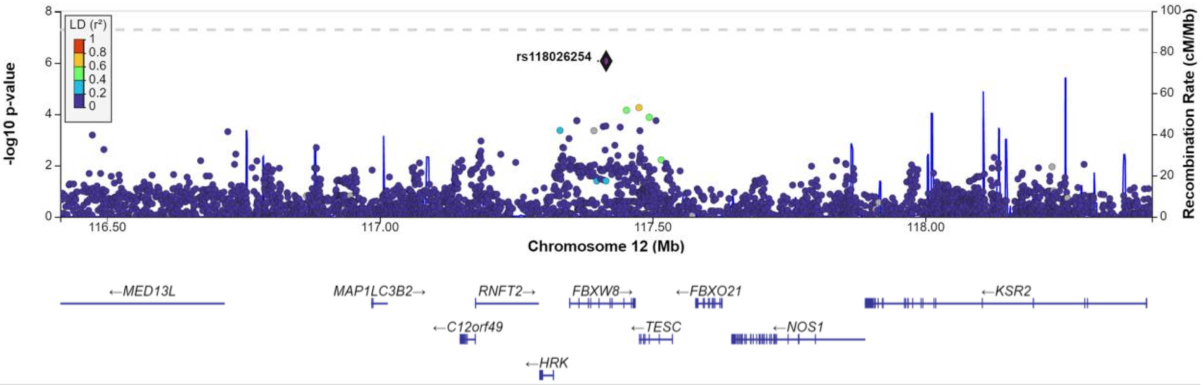

rs8022645 (14q23.3)

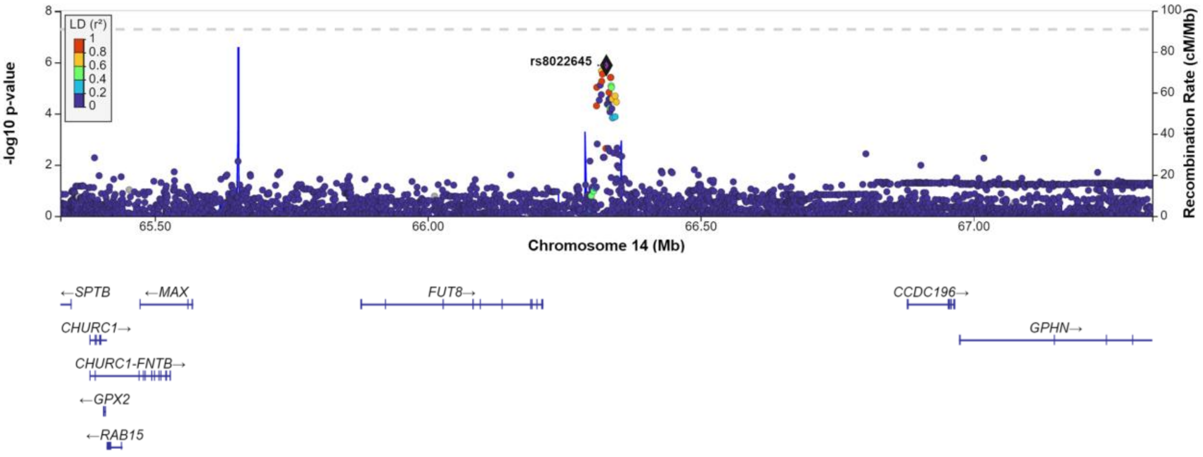

rs4989808 (15q26.1)

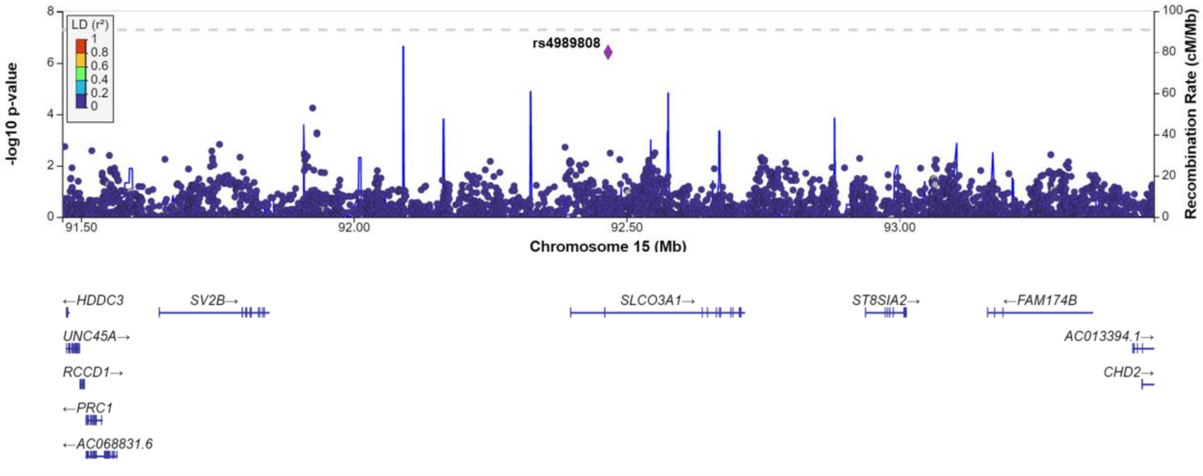

Figure S4. Forest plot of the validation analysis results for rs9508032 (at *FLT1*) and rs7967111 (at *BORCS5*) showing the odds ratios with 95% confidence intervals.

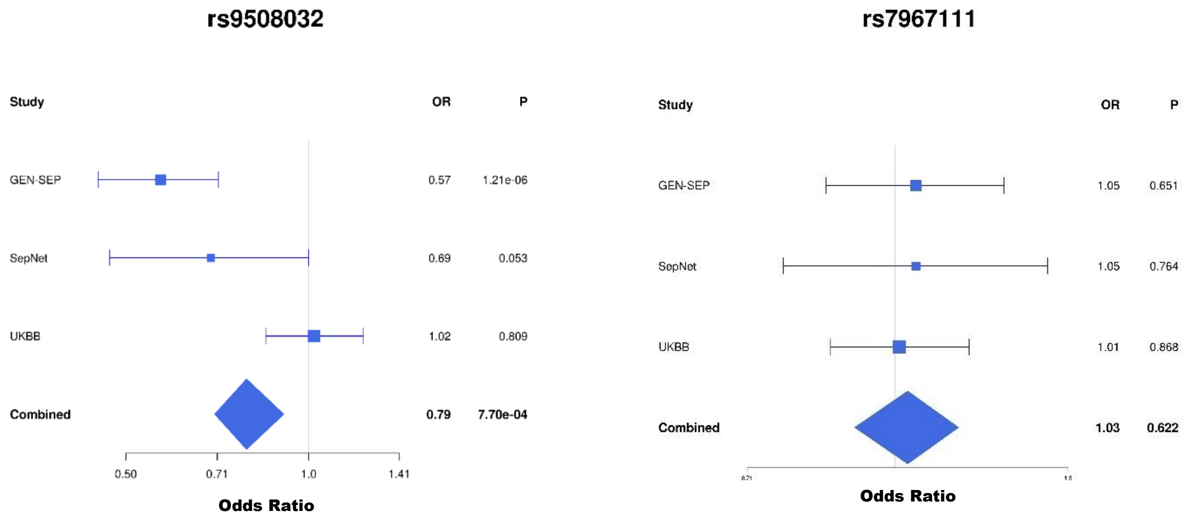

Figure S5. Results of the Bayesian fine mapping for the prioritised loci. The y-axis shows the transformed *p*-values (-$\log_{10}$ *p*-value), and the x-axis represents the chromosome positions (GRCh37/hg19). The orange diamond represents the sentinel variant of each region and the variants included in the credible sets are highlighted in green.

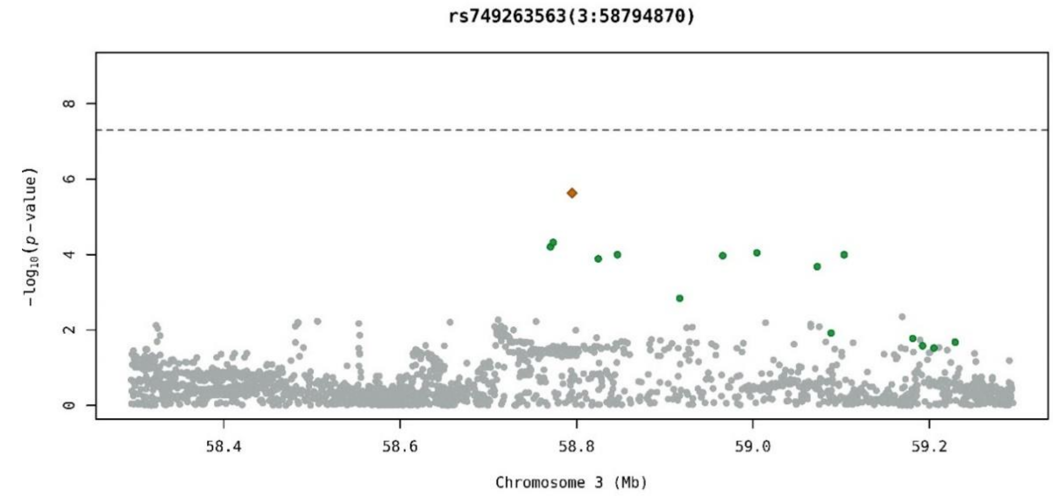

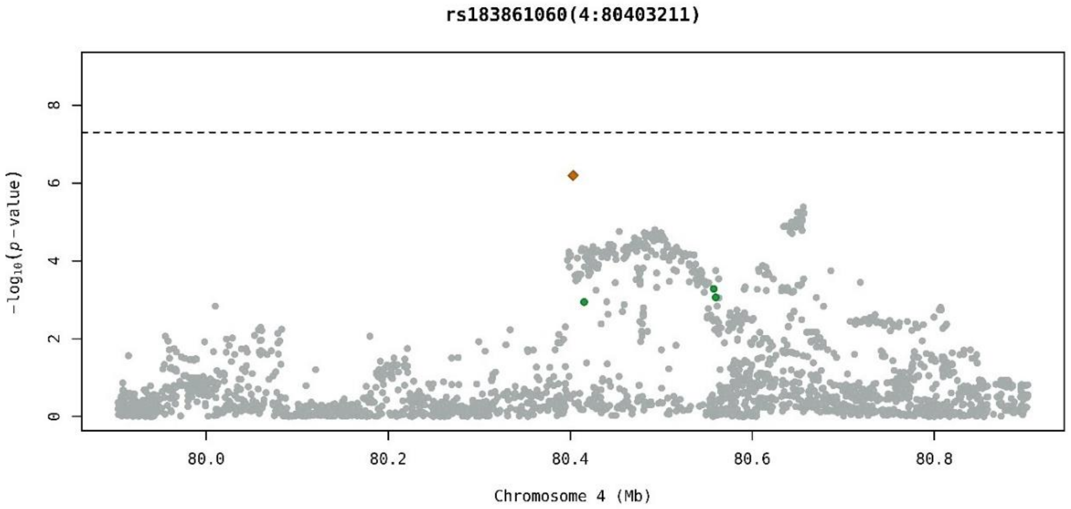

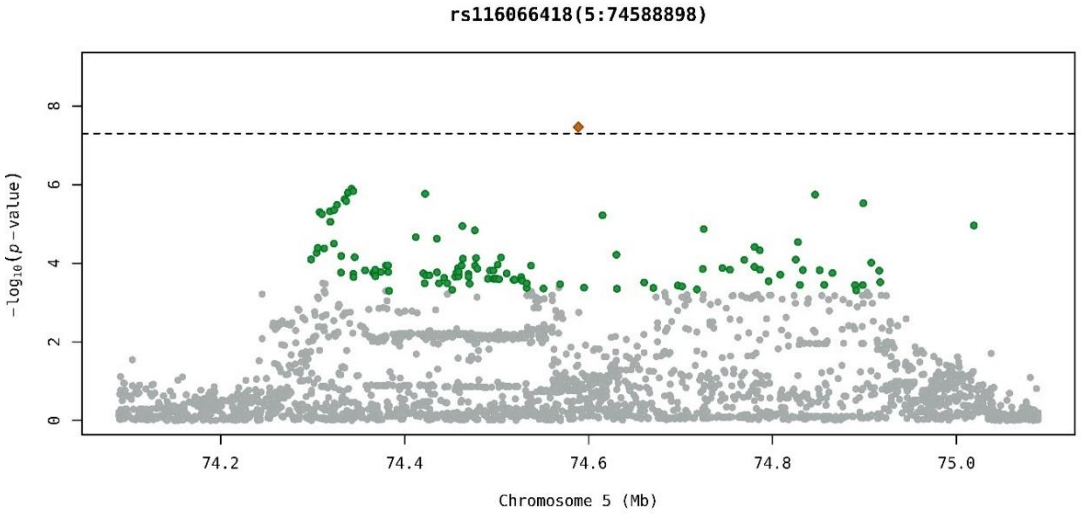

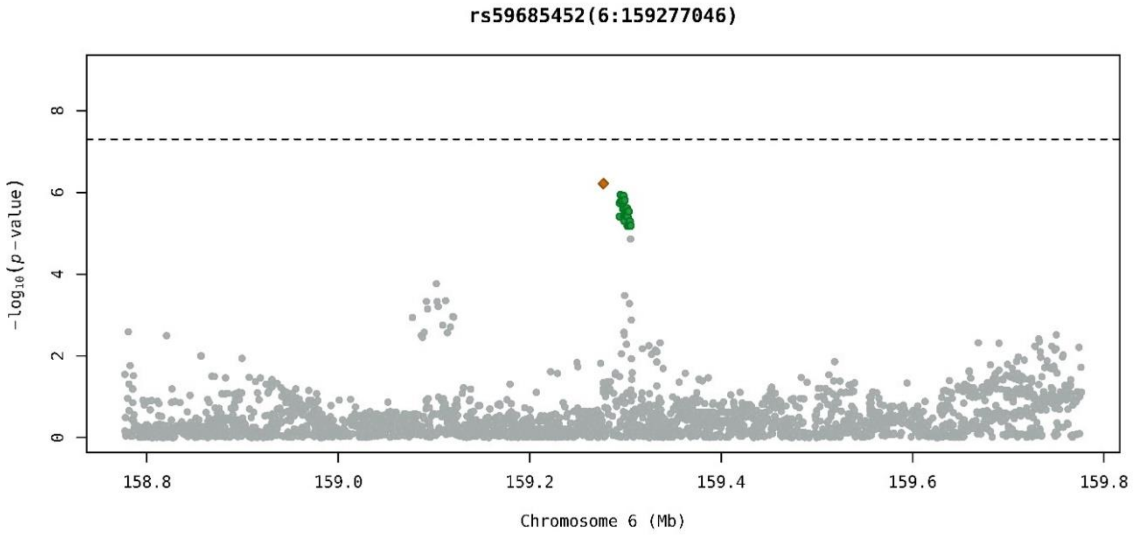
rs59685452(6:159277046)
−log10(p−value)
0
2
4
6
8
158.8
159.0
159.2
159.4
159.6
159.8
Chromosome 6 (Mb)

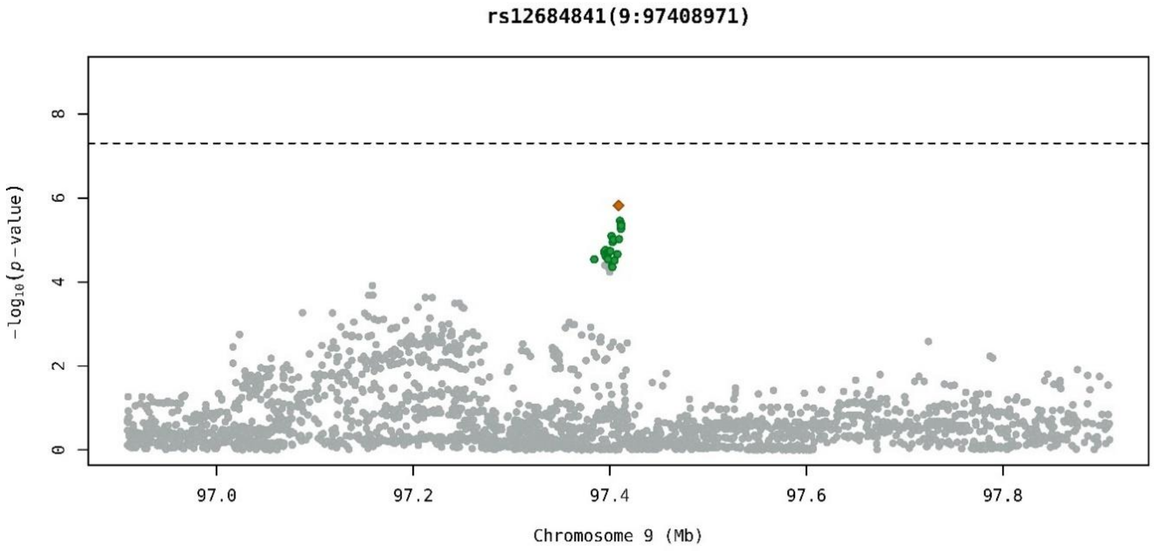
rs12684841(9:97408971)
−log10(p−value)
0
2
4
6
8
97.0
97.2
97.4
97.6
97.8
Chromosome 9 (Mb)

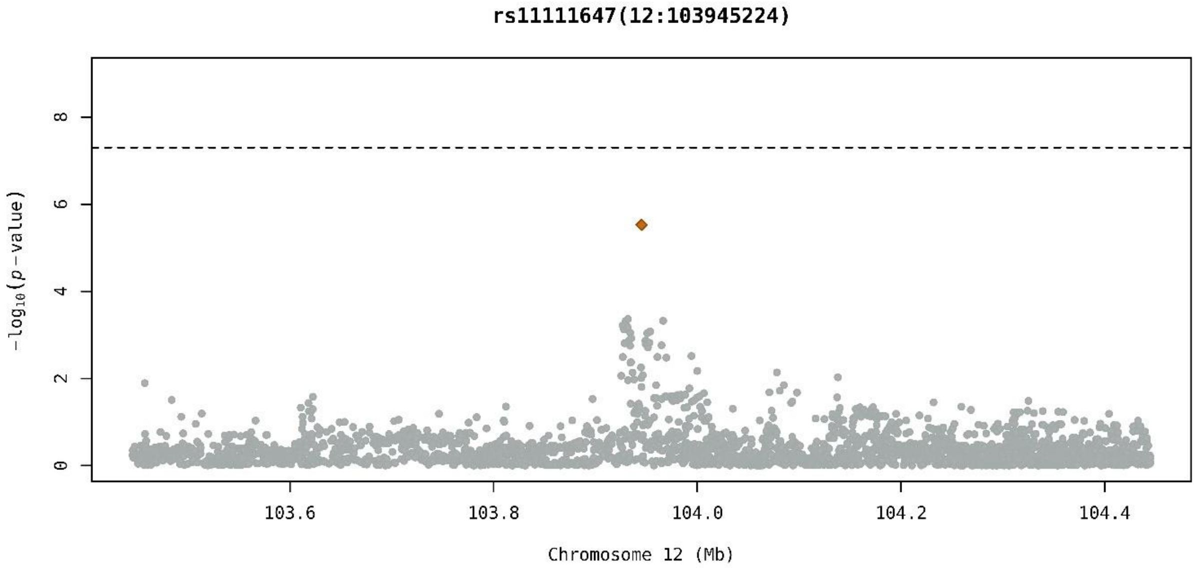
rs11111647(12:103945224)
−log10(p−value)
0
2
4
6
8
103.6
103.8
104.0
104.2
104.4
Chromosome 12 (Mb)

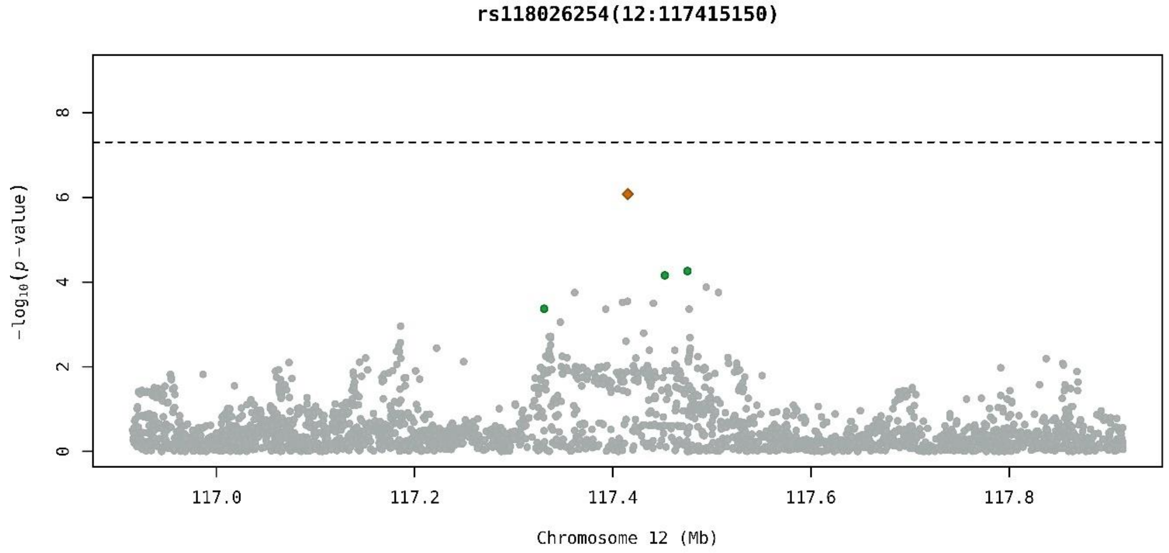
rs118026254(12:117415150)
-log10(p-value)
0
2
4
6
8
117.0
117.2
117.4
117.6
117.8
Chromosome 12 (Mb)

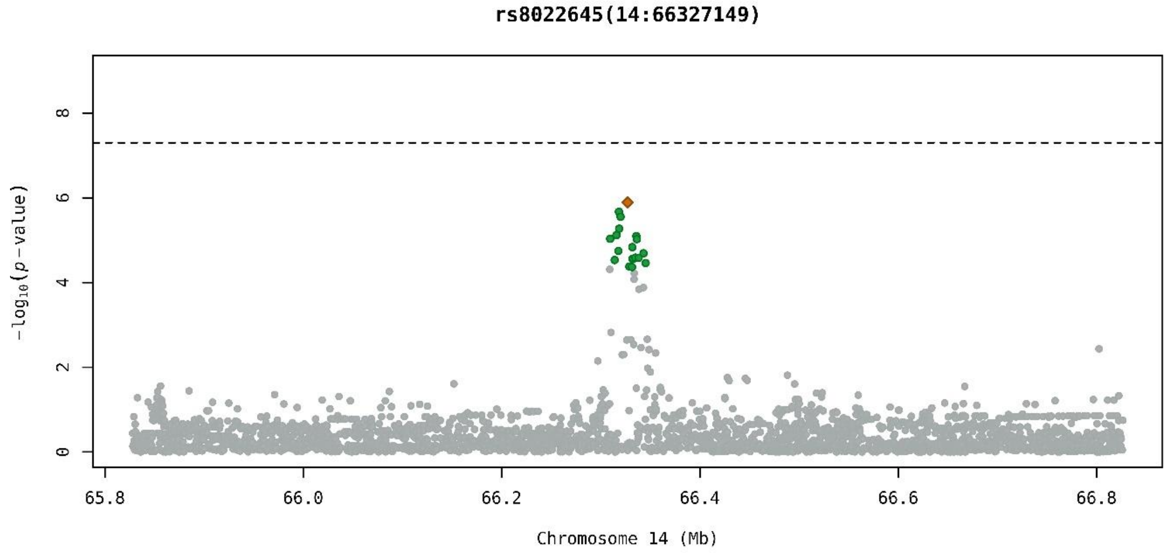
rs8022645(14:66327149)
-log10(p-value)
0
2
4
6
8
65.8
66.0
66.2
66.4
66.6
66.8
Chromosome 14 (Mb)

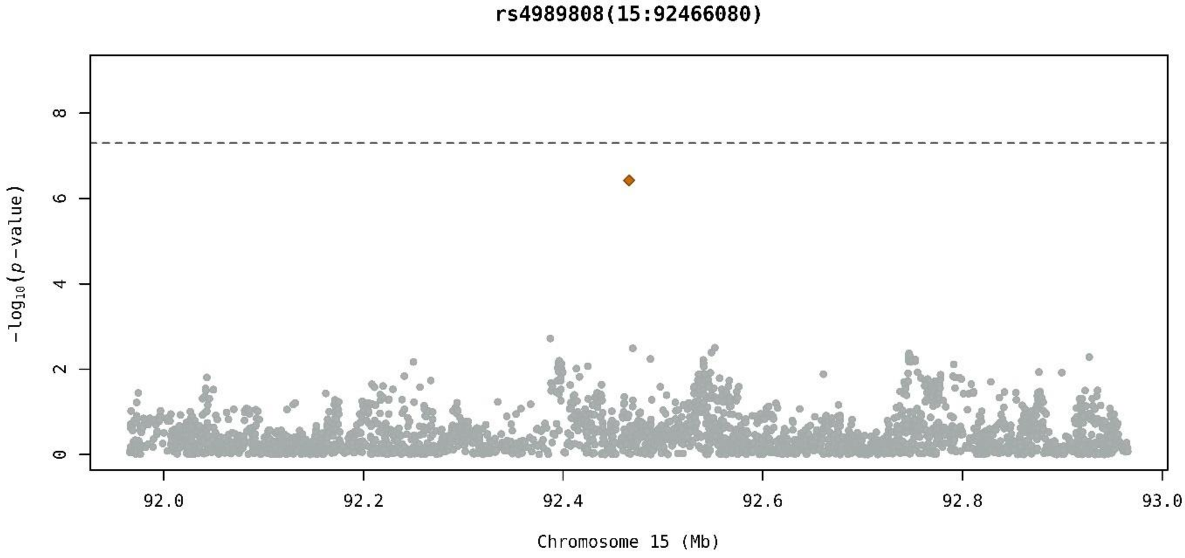
rs4989808(15:92466080)
-log10(p-value)
0
2
4
6
8
92.0
92.2
92.4
92.6
92.8
93.0
Chromosome 15 (Mb)

Supplementary Tables

Table S1. Main demographic and clinical characteristics of study participants

Locus	GEN-SEP		SepNet		UKBB	
	Controls (n=501)	Cases (n=304)	Controls (n=649)	Cases (n=91)	Controls (n=3,249)	Cases (n=321)
Sex (males)	301 (60.1%)	206 (67.8%)	379 (58.4%)	59 (64.8%)	1163 (35.8%)	115 (35.8%)
Mean age (years) ± SD	63.9 ± 15.3	62.4 ± 13.8	65.4 ± 14.1	62.1 ± 13.6	60.3 ± 6.9	59.4 ± 7.5
Severe infections*	501 (100%)	304 (100%)	649 (100%)	91 (100%)	2449 (75.4%)	242 (75.4%)
Sepsis	501 (100%)	304 (100%)	649 (100%)	91 (100%)	982 (30.2%)	97 (30.2%)
APACHE II (median) (P_{25}–P_{75})†	19 (14-24)	21 (17-27)	20 (16-24)	19 (16-23)	NA	NA

APACHE: Acute Physiology and Chronic Health Evaluation; NA: Not available data; P25: percentile 25; P75: percentile 75; SD: standard deviation. *This comprises pneumonia and/or sepsis

Table S2. ICD-10 codes used in the UK Biobank study

ARDS cause	ICD-10 Codes/ Data-Field 41270	Definition
Sepsis	A02.1	*Salmonella* septicaemia
	A20.7	Septicemic plague
	A22.7	Anthrax septicaemia
	A32.7	Listerial septicaemia
	A40	Streptococcal septicaemia
	A40.0	Septicaemia due to *Streptococcus*, group A
	A40.1	Septicaemia due to *Streptococcus*, group B
	A40.2	Septicaemia due to *Streptococcus*, group D
	A40.3	Septicaemia due to *Streptococcus pneumoniae*
	A40.8	Other streptococcal septicaemia
	A40.9	Streptococcal septicaemia, unspecified
	A41	Other septicaemia
	A41.0	Septicaemia due to *Staphylococcus aureus*
	A41.1	Septicaemia due to other specified *Staphylococcus*
	A41.2	Septicaemia due to unspecified *Staphylococcus*
	A41.3	Septicaemia due to *Haemophilus influenzae*
	A41.4	Septicaemia due to anaerobes
	A41.5	Septicaemia due to other Gram-negative organisms
	A41.8	Other specified septicaemia
	A41.9	Septicaemia, unspecified
	A42.7	Actinomycotic septicaemia
	B37.7	Candidal septicaemia
	R57.2	Septic shock
	R65.1	Systemic Inflammatory Response Syndrome of infectious origin with organ failure
Infections due to complications of surgical and medical care	T80.2	Infections following infusion, transfusion and therapeutic injection
	T81.4	Infection following a procedure, not elsewhere classified
	T82.6	Infection and inflammatory reaction due to cardiac valve prosthesis
	T82.7	Infection and inflammatory reaction due to other cardiac and vascular devices, implants and grafts
	T83.5	Infection and inflammatory reaction due to prosthetic device, implant and graft in urinary system
	T83.6	Infection and inflammatory reaction due to prosthetic device, implant and graft in genital tract

	T84.7	Infection and inflammatory reaction due to other internal orthopaedic prosthetic devices, implants and grafts
	T85.7	Infection and inflammatory reaction due to other internal prosthetic devices, implants and grafts
	T88.0	Infection following immunisation
Pneumonia	J10.0	Influenza with pneumonia, influenza virus identified
	J11.0	Influenza with pneumonia, virus not identified
	J12	Viral pneumonia, not elsewhere classified
	J13	Pneumonia due to *Streptococcus pneumoniae*
	J14	Pneumonia due to *Haemophilus influenzae*
	J15	Bacterial pneumonia, not elsewhere classified
	J16	Pneumonia due to other infectious organisms, not elsewhere classified
	J17	Pneumonia in diseases classified elsewhere
	J18	Pneumonia, organism unspecified
	J22	Unspecified acute lower respiratory infection
Pancreatitis	K85	Acute pancreatitis
Patients with ARDS associated to head, chest or other major injury (= Severe trauma)	T79	Certain early complications of trauma
	T00-T07	Injuries involving multiple body regions
	S02	Fracture of skull and facial bones
	S04	Injury of cranial nerves
	S06	Intracranial injury
	S07	Crushing injury of head
	S08	Traumatic amputation of part of head
	S097	Multiple injuries of head
	S22	Fracture of rib(s), sternum and thoracic spine
	S23.0	Traumatic rupture of thoracic intervertebral disc
	S24	Injury of nerves and spinal cord at thorax level
	S28	Crushing injury of thorax and traumatic amputation of part of thorax
	S29.7	Multiple injuries of thorax
Inhalation of harmful substances	W78	Inhalation of gastric contents
	T58	Toxic effect of carbon monoxide
	T59	Toxic effect of other gases, fumes and vapours
Poisoning	T36-T50	Poisoning by drugs, medicaments and biological substances
	X61	Intentional self-poisoning by and exposure to antiepileptic, sedative-hypnotic, antiparkinsonism and psychotropic drugs, not elsewhere classified

Table S3. Genetic ancestry of individuals in the UK Biobank study

Ancestry	%
African	1.25
Asian	3.12
Chinese	0.62
European	92.52
Mixed (black & white)	0.62
Mixed (others)	1.87

Table S4. Association results for the nine signals prioritised after excluding individuals without sepsis and/or pneumonia

			**All individuals (82.8% sepsis and/or pneumonia)** (716 cases/4,399 controls)			**Patients with sepsis and/or pneumonia only** (637 cases/3,599 controls)		
ID	Pos. (b37)	NEA/EA	OR (95%CI)	*p*-value	Direction*	OR (95%CI)	*p*-value	Direction*
rs749263563	3:58794870	TAGAC/T	2.66 (1.77, 4.00)	$2.34x10^{-6}$	?++	3.13 (1.99, 4.92)	$8.15x10^{-7}$	?++
rs183861060	4:80403211	A/G	2.95 (1.93, 4.52)	$6.39x10^{-7}$	+?+	2.42 (1.50, 3.90)	$2.74x10^{-4}$	+?+
rs116066418	5:74588898	T/A	2.54 (1.83, 3.54)	$3.43x10^{-8}$	+++	2.70 (1.88, 3.86)	$5.87x10^{-8}$	+++
rs59685452	6:159277046	G/C	2.12 (1.58, 2.86)	$6.10x10^{-7}$	+++	2.03 (1.46, 2.82)	$2.46x10^{-5}$	+++
rs12684841	9:97408971	A/G	1.63 (1.34, 1.99)	$1.51x10^{-6}$	+++	1.65 (1.33, 2.04)	$5.08x10^{-6}$	+++
rs11111647	12:103945224	G/A	1.40 (1.22, 1.62)	$2.97x10^{-6}$	+++	1.43 (1.22, 1.66)	$5.39x10^{-6}$	+++
rs118026254	12:117415150	G/C	2.18 (1.60, 2.96)	$8.38x10^{-7}$	+?+	2.05 (1.45, 2.90)	$4.85x10^{-5}$	+?+
rs8022645	14:66327149	C/T	1.40 (1.22, 1.61)	$1.28x10^{-6}$	+++	1.45 (1.25, 1.68)	$1.03x10^{-6}$	+++
rs4989808	15:92466080	A/G	2.66 (1.82, 3.87)	$3.77x10^{-7}$	+?+	3.19 (2.13, 4.77)	$1.56x10^{-8}$	+?+

CI: confidence interval, EA: effect allele, NEA: non-effect allele, OR: Odds ratio, Pos.: position. *Order for effect Direction is: GEN-SEP – SepNet – UKBB (+ means beta >0 and ? means missing data).

Table S5. Association results for rs9508032 (at *FLT1*) and rs7967111 (at *BORCS5*) after excluding individuals without sepsis and/or pneumonia

			**All individuals (82.8% sepsis and/or pneumonia)** (716 cases/4,399 controls)			**Patients with sepsis and/or pneumonia only** (637 cases/3,599 controls)		
ID	Pos. (b37)	NEA/EA	OR (95%CI)	*p*-value	Direction*	OR (95%CI)	*p*-value	Direction*
rs9508032	13:28995940	C/T	0.79 (0.70, 0.91)	$7.70x10^{-4}$ #	---+	0.76 (0.66, 0.87)	$1.35x10^{-4}$ ¥	---
rs7967111	12:12601953	G/A	1.03 (0.91, 1.16)	0.622	+++	1.03 (0.90, 1.16)	0.70	++-

CI: confidence interval, EA: effect allele, NEA: non-effect allele, OR: Odds ratio, Pos.: position. *Order for effect Direction is: GEN-SEP – SepNet – UKBB (+ means beta >0; – means beta <0). #Non-significant in UKBB (p=0.809); ¥Non-significant in UKBB (p=0.955).

Table S6. Bayesian fine mapping and SNP deleteriousness assessment

Locus	Credible set	Highest PP from the credible set	Sentinel variant			Highest phenotypic impact variant*			
			rsid	PP	CADD score	rsid	r^2	PP	CADD score
**3p14.2**	15	0.218	rs749263563	0.218	0.39	rs80308704	0.89	0.081	17.80
**4q21.21**	4	0.735	rs183861060	0.735	6.72	rs183861060	1.00	0.735	6.72
**5q13.3**	119	0.090	rs116066418	0.060	3.18	rs6893216	0.18	0.002	16.66
**6q25.3**	33	0.079	rs59685452	0.079	0.87	rs75891667	0.92	0.032	14.33
**9q22.32**	28	0.150	rs12684841	0.150	1.46	rs73523269	0.96	0.056	15.78
**12q23.3**	1	0.972	rs11111647	0.972	5.44	rs11111647	1.00	0.972	5.44
**12q24.22**	4	0.795	rs118026254	0.795	0.31	rs117510704	0.41	0.033	5.67
**14q23.3**	19	0.231	rs8022645	0.231	2.15	rs12896836	0.39	0.018	13.71
**15q26.1**	NA	NA	rs4989808	NA	7.77	rs4989808	1.00	NA	7.77

CADD: combined annotation dependent depletion v.1.6; PP: Posterior probabilities; r^2: linkage disequilibrium between the sentinel variant and the SNP with the highest phenotypic impact in the locus. *Prioritised SNP based on the top-ranked CADD score for each locus.

Table S7. Highest phenotypic impact SNPs follow-up: first-stage meta-analysis association results, gene expression and PheWAS

Sentinel variant [locus]	Highest phenotypic impact SNP*	chr:position (GRCh37/hg19)	NEA/EA	OR (95%CI)	*p*-value	Nearest gene/s	eQTLs (p<6.25x10^{-3}) #	PheWAS (p<1x10^{-5})
rs749263563, [**3p14.2**]	rs80308704 (MAF= 0.023)	3:58965636	G/T	1.88 (1.37, 2.59)	1.07x10^{-4}	*CFAP20DC*	NA	NA
rs183861060, [**4q21.21**]	rs183861060 (MAF=0.025)	4:80403211	A/G	2.95 (1.93, 4.52)	6.39x10^{-7}	*GK2*	NA	NA
rs116066418, [**5q13.3**]	rs6893216 (MAF=0.114)	5:74442964	T/C	1.38 (1.16, 1.65)	2.35x10^{-4}	*ANKRD31*	+ *POC5* (Cultured fibroblasts, Thyroid, EBV-transformed lymphocytes).<br>H4 = <5%; H3 = 87%<br><br>+ *ANKDD1B* (Artery-Tibial).<br>H4 = 71.50% [colocalise]; H3 = 25.20%<br><br>+ *ANKDD1B* (Artery-Aorta).<br>H4 = 28.20%; H3= 60.30%	Low density lipoprotein cholesterol levels, Total cholesterol levels, Apolipoprotein B levels, Statin medication, Platelet count, Mean platelet volume, C-reactive protein levels, Disorders of lipoid metabolism.
rs59685452, [**6q25.3**]	rs75891667 (MAF=0.020)	6:159301294	A/G	2.05 (1.52, 2.77)	2.43x10^{-6}	*EZR*	NA	NA
rs12684841, [**9q22.32**]	rs73523269 (MAF=0.070)	9:97411359	T/C	1.59 (1.30, 1.94)	5.44x10^{-6}	*FBP1, AOPEP*	- *FBP1* (Oesophagus-Mucosa).<br>H4 = 88.80% [colocalise]; H3 = 5.41%<br><br>+ *MFSD14B* (Whole Blood)<br>H4 = 5.36%; H3 = 74.30%	NA
rs11111647, [**12q23.3**]	rs11111647 (MAF=0.191)	12:103945224	G/A	1.40 (1.22, 1.62)	2.97x10^{-6}	*STAB2*	NA	NA

**Sentinel variant [locus]**	**Highest phenotypic impact SNP***	**chr:position (GRCh37/hg19)**	**NEA/EA**	**OR (95%CI)**	***p*-value**	**Nearest gene/s**	**eQTLs (p<6.25x10^{-3}) #**	**PheWAS (p<1x10^{-5})**
rs118026254 [**12q24.22**]	rs117510704 (MAF=0.067)	12:117330700	T/C	1.56 (1.22, 1.99)	4.27x10^{-4}	*HRK* / *FBXW8*	NA	NA
rs8022645 [**14q23.3**]	rs12896836 (MAF=0.596)	14:66332007	C/T	1.29 (1.15, 1.45)	2.74x10^{-5}	*FUT8* / *CCDC196*	NA	NA
rs4989808 [**15q26.1**]	rs4989808 (MAF=0.974)	15:92466080	A/G	2.66 (1.82, 3.87)	3.77x10^{-7}	*SLCO3A1*	NA	NA

*Prioritised SNP based on the top-ranked CADD score for each locus. # Increased (+) or decreased (-) gene expression of significant eQTLs based on a Bonferroni-corrected p<6.25x10^{-3}. Directions of effect refer to the ARDS risk allele. The H4 values inform the posterior probability of colocalisation between the ARDS risk signal and the gene expression eQTL signal in the tissues stated. A H4>70% indicates that both traits are associated and share a single causal variant, while A H3>70% indicates that both traits are associated, but with different causal variants. CADD: combined annotation dependent depletion v.1.6, chr: chromosome, CI: confidence interval, EBP: Epstein-Barr Virus; eQTLs: expression quantitative locus, based on Genotype-Tissue Expression (GTEx) project V8 (https://www.gtexportal.org/home/); MAF: minor allele frequency based on gnomAD v2.1.1 data from non-Finnish European individuals; NEA/EA: non-effect allele/effect allele; OR: odds ratio, PheWAS: Phenome-wide association study results based on a Bonferroni-corrected p-value<1x10^{-5} for the prioritised SNPs (https://genetics.opentargets.org/).
